# Electrolyte‐Replacement‐Free Continuous Electrocatalytic Desalination Coupled With CO_2_ Reduction at Record Throughput and Low Cost

**DOI:** 10.1002/anie.9124699

**Published:** 2026-05-01

**Authors:** Man Liang, Pucheng Duan, Minzhang Li, Zhefei Wu, Lu Guo, Afzalshoh Qahramon Zarifzoda, Chengli Rong, Fuming Chen, Yuan Chen

**Affiliations:** ^1^ State Key Laboratory of Tropic Ocean Engineering Materials and Materials Evaluation School of Chemistry and Chemical Engineering Hainan University Haikou China; ^2^ Guangdong Provincial Key Laboratory of Quantum Engineering and Quantum Materials Guangdong Engineering Technology Research Center of Efficient Green Energy and Environment Protection Materials School of Electronic Science and Engineering (School of Microelectronics) South China Normal University Foshan China; ^3^ Yunnan‐Malaya Institute School of Engineering Yunnan University Kunming China; ^4^ S.U. Umarov Physical‐Technical Institute of the National Academy of Sciences of Tajikistan Dushanbe Tajikistan; ^5^ School of Chemical and Biomolecular Engineering The University of Sydney Darlington New South Wales Australia

**Keywords:** circulating electrolyte, CO_2_ electroreduction, cobalt phthalocyanine, electrocatalytic desalination

## Abstract

Integrating seawater desalination with electrocatalytic reactions offers an attractive pathway to address freshwater scarcity and reduce emissions simultaneously. However, practical implementation has been impeded by slow desalination, electrolyte degradation, and frequent electrolyte replacement, all of which increase operating costs and limit scalability. Here, we report a continuous electrocatalytic desalination system driven by CO_2_ electroreduction that fundamentally eliminates the need for electrolyte replacement via a self‐balancing circulating electrolyte architecture. A five‐chamber cell incorporating a salt‐concentration chamber and an interconnected anolyte–catholyte loop enables sustained ion transport while suppressing byproduct accumulation. Coupled with a highly active nanorod cobalt phthalocyanine/carboxylated carbon nanotube catalyst, the device delivers high current density and stable CO_2_‐to‐CO conversion. Using natural seawater, the cell achieves an ultrafast salt removal rate of 1592.8 µg cm^−^^2^ min^−^^1^ over 90 h of continuous operation without electrolyte replacement, representing one of the highest values reported for electrocatalytic desalination. Simultaneously, CO production proceeds with a Faradaic efficiency of 95.5%–96.4% and a production rate exceeding 683 µmol cm^−^^2^ h^−^^1^. The desalinated water reaches potable standards with >99% salt removal, while techno‐economic analysis reveals a drastic reduction in daily electrolyte costs. This work establishes a scalable strategy for high‐throughput, low‐cost desalination integrated with CO_2_ valorization.

## Graphical abstract

A continuous electrocatalytic desalination system powered by CO_2_ electroreduction that eliminates electrolyte replacement through a self‐balancing circulating design. Using a five‐chamber architecture and an efficient cobalt‐based catalyst, it achieves ultrafast salt removal, high CO production efficiency, and stable long‐term operation, offering a scalable, low‐cost solution for desalination and carbon utilization.

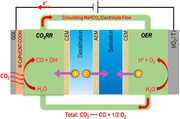

## 1 Introduction

Freshwater scarcity poses a substantial threat to human societies and ecosystems worldwide [1, 2]. Seawater desalination is a critical intervention to mitigate this challenge [3, 4]. However, conventional desalination technologies are energy‐intensive and predominantly powered by fossil fuels, thereby exacerbating greenhouse gas emissions [5, 6, 7]. Despite considerable research efforts to develop alternative desalination technologies, their widespread deployment remains limited by unsatisfactory performance and high capital and operational costs [8, 9, 10, 11]. Electrocatalytic desalination has recently emerged as a potential strategy to address freshwater scarcity while simultaneously reducing greenhouse gas emissions [12, 13, 14, 15, 16, 17]. In a typical electrocatalytic desalination process, electrochemical reactions generate ionic concentration gradients in the electrolyte, producing an electrochemical potential that drives the transport of salt ions (e.g., Na^+^ and Cl^−^) across selectively permeable ion‐exchange membranes (IEMs), thereby achieving desalination [18, 19]. However, two challenges hinder the practical implementation of electrocatalytic desalination processes. First, desalination rates are generally slow, significantly slower than other desalination technologies such as reverse osmosis (RO) or electrodialysis (ED) [12, 13, 20]. Second, the accumulation of electrochemical reaction byproducts in electrolytes necessitates frequent electrolyte replacement, increasing operational costs and severely limiting scalability.

The electrochemical CO_2_ reduction reaction (CO_2_RR) is a sustainable route to convert the greenhouse gas CO_2_ into value‐added chemicals and fuels [21, 22, 23, 24, 25]. In particular, the two‐electron‐transfer reduction gas product, carbon monoxide (CO), can be readily separated, and CO_2_RR toward CO production is currently considered the most viable for large‐scale commercialization [26, 27, 28, 29, 30]. We previously demonstrated that CO_2_RR can drive electrocatalytic desalination [31]. However, the desalination rate has been limited by ion flux across IEMs, controlled by the catalytic activity of CO_2_RR electrocatalysts [31, 32]. Furthermore, the accumulation of NaOH byproducts from CO_2_RR in the electrolyte requires frequent electrolyte replacement, which remains a significant obstacle to practical implementation.

Herein, we report the use of an efficient and durable metal phthalocyanine‐based electrocatalyst deposited onto a gas diffusion layer (GDL) as the working electrode to increase current density, thus accelerating desalination. We also design a new five‐chamber electrocatalytic desalination device featuring a salt‐collection chamber and a circulating electrolyte system with interconnected anolyte and catholyte chambers for Na^+^ removal and to suppress OH^−^ accumulation, thereby avoiding frequent electrolyte replacement. This device achieves an ultrafast salt removal rate (*SRR*) of 1592.8 µg cm^−^^2^ min^−^^1^, representing a 50% improvement over the previously reported best value, during a long‐term stability test of approximately 90 h using natural seawater without electrolyte replacement. The catalyst delivers a high Faradaic efficiency of CO (*FE*_CO_) exceeding 95.5%, with an average CO production rate (*PR*_CO_) above 683 µmol cm^−^^2^ h^−^^1^. Furthermore, salt removal efficiency (SRE) for natural seawater exceeded 99%, meeting potable water standards. The economic viability of the desalination cell is further supported by a comprehensive techno‐economic analysis (TEA).

## 2 Results and Discussion

### 2.1 Seawater Desalination Device With Circulating Electrolyte

**FIGURE 1:**
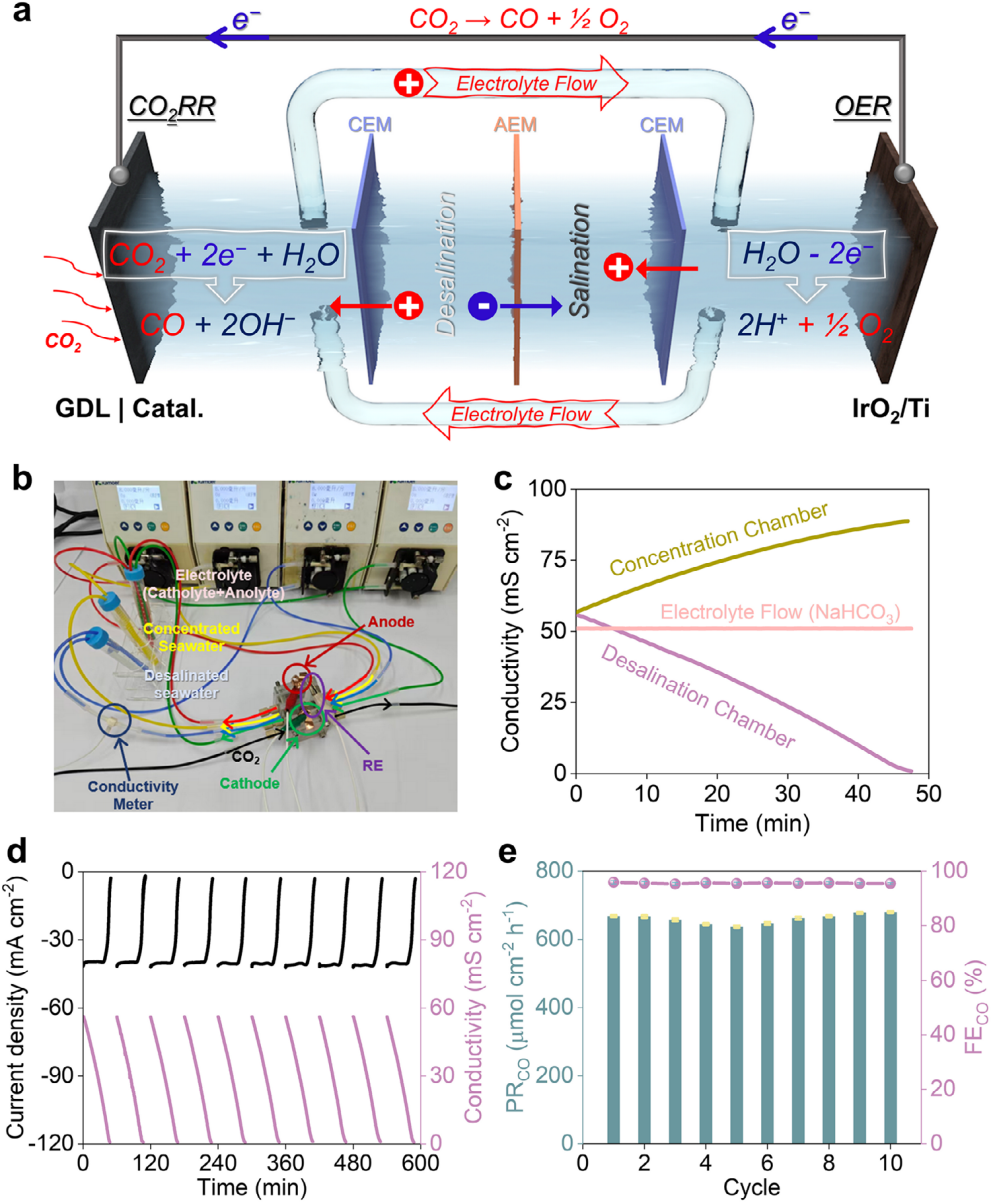
(a) Schematic illustration of an electrocatalytic desalination device with five chambers that enables a continuous circulating electrolyte flow; (b) a photo of the device in operation; (c) the changes of ionic conductivity in salt concentration and desalination chambers and electrolyte flow during device operation; (d) *I*–*t* curves and the corresponding ionic conductivity changes during 10 cycles of seawater desalination without electrolyte refreshment, and (e) the corresponding *FE*_CO_ and *PR*_CO_.

We hypothesize that NaOH byproducts can be consumed through proper electrolyte mixing across different chambers, thereby eliminating the need for electrolyte replacement. As illustrated in Figure 1a, the new electrocatalytic desalination device comprises five functional chambers: a CO_2_ diffusion chamber, a cathode chamber for CO_2_RR, an anode chamber for the oxygen evolution reaction (OER), a desalination chamber for hosting seawater, and a salt‐concentrating chamber for ion collection and removal. The desalination and salt‐concentrating chambers are positioned at the center of the device and are separated by an anion‐exchange membrane (AEM). These central chambers are flanked by cathode and anode chambers, separated by two cation‐exchange membranes (CEMs) on each side. A photograph of the assembled device in operation is shown in Figure 1b.

Compared with previously reported electrocatalytic desalination systems (Figure S1) [17, 31, 32], this device incorporates two key design innovations. First, a dedicated salt‐concentrating chamber is introduced to enable continuous ion accumulation and removal. Second, the catholyte and anolyte chambers are interconnected via a circulating electrolyte loop. Together, these features establish a closed cation‐transport cycle that enables continuous Na^+^ removal while preventing the accumulation of electrochemical byproducts, thereby eliminating the need for electrolyte replacement.

During operation, anions (e.g., Cl^−^ and SO_4_^2^^−^) in the desalination chamber migrate directly through the AEM into the salt‐concentrating chamber. In contrast, cations (e.g., Na^+^ and K^+^) migrate across two CEMs: they first enter the cathode chamber and subsequently reach the salt‐concentrating chamber through the circulating electrolyte loop. At the cathode, CO_2_RR occurs at a gas diffusion electrode, where CO_2_ is supplied through the gas chamber on the outer side of the GDL. The inner surface of the GDL is coated with the metal phthalocyanine‐based catalyst that selectively converts CO_2_ to CO. The anode consists of a commercial Ti/IrO_2_ electrode to catalyze the OER. Notably, Cl^−^ ions from seawater are effectively blocked from entering the cathode/anode chambers by the membrane configuration, thereby suppressing chlorine evolution side reactions and ensuring stable, efficient operation. As a result, this device design enables simultaneous stable seawater desalination and efficient electrochemical CO_2_RR without electrolyte replacement. The overall electrochemical reactions are as follows:

$$\mathrm{Total}\mathrm{reaction}:\;CO_{2}\to \mathrm{CO}+1/2\;\;O_{2}$$

$$\mathrm{Cathode}\;\left(CO_{2}\mathrm{RR}\right):\;CO_{2}+H_{2}O+2\;e^{-}\to \mathrm{CO}+2\;OH^{+}$$

$$\mathrm{Anode}\;\left(\mathrm{OER}\right):\;H_{2}O-2\;e^{-}\to 1/2\;O_{2}+2\;H^{+}$$

As shown in Figure 1c, coupled seawater desalination and CO_2_RR were performed using natural seawater (conductivity ≈ 56 mS cm^−^^1^, salinity ≈ 35,000 ppm) under optimized conditions (−1.3 V vs. RHE, 1 M NaHCO_3_). The seawater in the desalination chamber was consistently desalinated within 48 min, as evidenced by a decrease in conductivity to below 1 mS cm^−^^1^, while the current density remained stable at values exceeding 40 mA cm^−^^2^ (Figure 1d). In contrast, the conductivity of the salt‐concentrating chamber increased continuously, confirming effective ion migration from the desalination chamber. Notably, the conductivity of the circulating electrolyte remained nearly constant at approximately 51 mS cm^−^^1^ throughout the process, indicating that the electrolyte composition was well maintained. Continuous pH monitoring (Figure S2) of electrolytes in different chambers during desalination shows that the circulating electrolyte chamber maintained a stable near‐neutral pH of 8.31–8.35 during operation, validating our design principle that H^+^ and OH^−^ generated at the electrodes are effectively neutralized within the electrolyte loop. Figure 1d further presents cyclic desalination experiments over 10 consecutive batches conducted without refreshing electrolytes. Throughout these cycles, the cathodic *FE*_CO_ remained above 95.3%, while hydrogen evolution was effectively suppressed. The Faradaic efficiency of H_2_ production ($FE_{H_{2}}$) is less than 5% (Figure S3). In addition, *PR*_CO_ consistently exceeds 637 µmol cm^−^^2^ h^−^^1^ (Figure 1e), demonstrating the excellent continuity and long‐term operational stability of the device.

### 2.2 Synthesis and Characterization of N‐CoPc/CNT‐COOH Electrocatalyst

**FIGURE 2:**
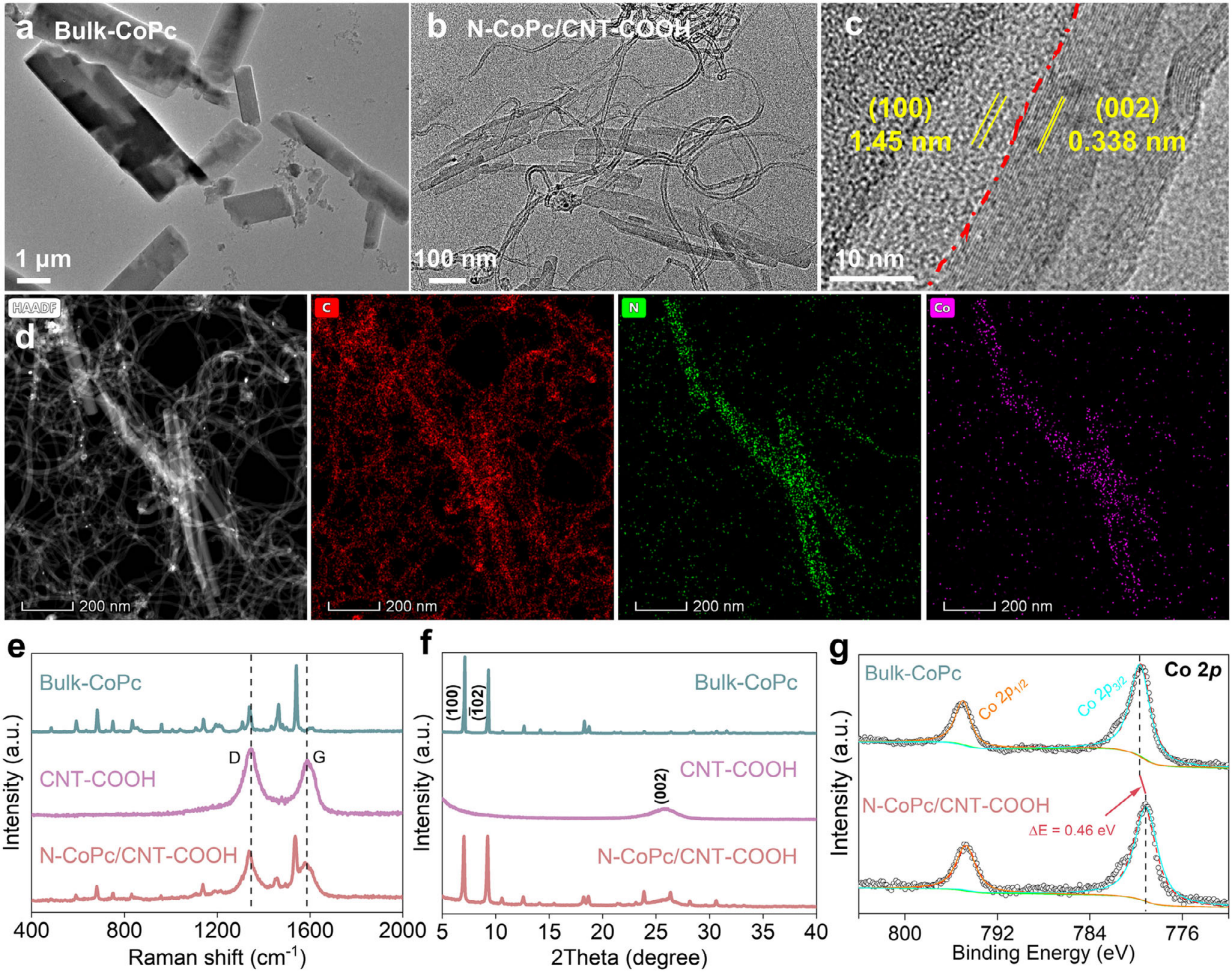
(a) A TEM image of bulk CoPc; (b) TEM and (c) HR‐TEM images of N‐CoPc/CNT‐COOH; (d) a high‐angle annular dark‐field TEM image and the corresponding EDS element mappings of N‐CoPc/CNT‐COOH; (e) Raman spectra and (f) XRD patterns of bulk CoPc, CNT‐COOH, and N‐CoPc/CNT‐COOH; (g) XPS spectra of Co 2*p* regions of bulk CoPc and N‐CoPc/CNT‐COOH.

The device's efficiency relies on the catalytic activity of its CO_2_RR electrocatalyst. Metal phthalocyanines, which contain abundant M–N_4_ active sites and are low‐cost, exhibit excellent chemical stability and have been explored as CO_2_RR electrocatalysts [33, 34, 35, 36, 37]. Previous studies show that uniformly anchoring individual metal phthalocyanine molecules to conductive substrates results in high selectivity for methanol production [38, 39, 40, 41, 42]. In contrast, several other studies show that nanostructures formed by the self‐assembly of metal phthalocyanines are more efficient for CO_2_RR to produce CO [39, 41, 42]. For example, aggregated Co octaethoxyphthalocyanine delivered a CO Faradaic efficiency (*FE*_CO_ ≥ 95%) with an industrially relevant CO partial current density (*j*_CO_ ≥ 300 mA cm^−2^) in a flow‐cell configuration [43]. We hypothesize that aggregated nanostructures of cobalt phthalocyanine (CoPc) can deliver stable high currents to enable rapid desalination. We synthesized CoPc nanorods (N‐CoPc) by a simple precipitation method and wrapped them with carboxylated multi‐walled carbon nanotubes (CNT‐COOH). CNT‐COOH was used instead of pristine CNTs to ensure their good dispersion in solvents and attachment to N‐CoPc to increase the catalyst's hydrophilicity. The synthesis details are described in the Supporting Information and illustrated in Figure S4, with a mass ratio of CoPc to CNT‐COOH of 2:1. Catalyst characterization by transmission electron microscopy (TEM) revealed the catalyst's morphological features. Bulk CoPc contains micrometer‐sized flake‐like aggregates (Figure 2a) due to strong π–π stacking among its conjugated planes. In contrast, CoPc self‐assembles into nanorods (N‐CoPc) that intimately intertwine with CNT‐COOH (Figure 2b). CNT‐COOH forms a vine‐like conductive network that maximizes electron and mass transfer to active sites on N‐CoPc [42]. The high‐resolution TEM (HR‐TEM) image of N‐CoPc/CNT‐COOH in Figure 2c reveals well‐defined lattice fringes with a spacing of 0.338 nm, corresponding to the (002) plane of CNTs, accompanied by comparatively vague but sequential fringes (*d* spacing = 1.45 nm) mainly attributable to the (100) planes in N‐CoPc. The spatial distribution of constituent elements (C, N, Co), obtained from energy‐dispersive X‐ray spectroscopy (EDX) mapping (Figure 2d), confirms the structure of N‐CoPc/CNT‐COOH.

Raman spectroscopy was employed to characterize CoPc, CNTs, and N‐CoPc/CNT‐COOH. As presented in Figure 2e, the spectra of CNTs and N‐CoPc/CNT‐COOH display the characteristic D band (∼1345 cm^−^^1^) and G band (∼1585 cm^−^^1^) from CNTs, corresponding to defects and vibrational modes of graphitic carbon [44]. N‐CoPc/CNT‐COOH exhibits a sharp peak at 1535 cm^−^^1^ and some discernible signals between 500 and 1200 cm^−^^1^, which match the prominent characteristic peaks of bulk CoPc, confirming the successful incorporation of CoPc into N‐CoPc/CNT‐COOH. XRD patterns of CoPc, CNTs, and N‐CoPc/CNT‐COOH are shown in Figure 2f. A broad diffraction peak near 26° (2*θ*) is observed, corresponding to the (002) crystallographic plane of graphitic carbon in CNTs [42, 45]. The XRD pattern of CoPc shows intense peaks at 7.1° and 9.3° (2*θ*), which are characteristic of the (100) and ($\bar{1}$02) planes and confirm the *β*‐phase MPc structure [41]. The peaks observed in N‐CoPc/CNT‐COOH align with the characteristic features of CoPc, verifying successful hybridization without destroying the intrinsic structures of CoPc. Ultraviolet‐visible‐near‐infrared (UV–vis–NIR) and Fourier transform infrared (FTIR) spectra provide additional structural information. CNT‐COOH shows low absorption in the visible region (Figure S5). In contrast, N‐CoPc/CNT‐COOH exhibits a redshifted Q‐band compared to CoPc, suggesting the formation of aggregated CoPc nanorods during the precipitation process and their immobilization in N‐N‐CoPc/CNT‐COOH‐COOH [46]. As shown in the FTIR spectra (Figure S6), both N‐CoPc/CNT‐COOH and CNT‐COOH exhibit a distinct C═C stretching vibration peak (1633 cm^−^^1^) arising from the skeleton of CNTs [47]. The bulk CoPc spectrum is characterized by typical phthalocyanine vibrations: skeletal C─N/C═C stretches (1550–1250 cm^−^^1^) and C–H bends (1200–700 cm^−^^1^) [48]. The identical set of peaks observed in N‐CoPc/CNT‐COOH corroborates the structural integrity of the CoPc moiety after immobilization.

XPS analysis was conducted to determine the surface composition and chemical states. As shown in the survey spectra (Figure S7a), the elements C, N, O, and Co are identified in both the bulk CoPc and N‐CoPc/CNT‐COOH. The origin of the O 1*s* peak is postulated to be a combination of adsorbed atmospheric species (oxygen/water) and the carboxyl functional groups from CNT‐COOH. High‐resolution XPS of the Co 2*p* region (Figure 2g) confirms the Co(II) state in the Co‐N_4_ structure of bulk CoPc, with binding energies of 779.65 eV (Co 2*p*_3/2_) and 795.02 eV (Co 2*p*_1/2_) [33]. In contrast, the Co 2*p* peaks in N‐CoPc/CNT‐COOH are negatively shifted by approximately 0.46 eV. This shift indicates a modification of the Co electronic environment, consistent with noncovalent π–π stacking between the phthalocyanine and the CNT‐COOH surface [32, 42]. As shown in Figure S7b, the characteristic N species of the phthalocyanine ring in bulk CoPc are identified at 397.46 eV (pyridinic‐N) and 399.50 eV (pyrrolic‐N/Co‐N). For N‐CoPc/CNT‐COOH, the pyrrolic‐N/Co‐N peak shifts negatively to 399.26 eV. This shift suggests an elevated electron density at the Co‐N_4_ site, presumably from electron transfer from CNT‐COOH. Such an electron‐rich Co center promotes CO_2_ adsorption and activation, potentially boosting the CO_2_RR performance [17, 49, 50].

### 2.3 Performance of N‐CoPc/CNT‐COOH in the Desalination Cell

We first compared the electrochemical performance of bulk CoPc and N‐CoPc/CNT‐COOH for CO_2_RR in a flow‐cell configuration using a neutral electrolyte (1 M NaHCO_3_) (Figure S8). Linear sweep voltammetry (LSV) measurements revealed that N‐CoPc/CNT‐COOH achieved higher current densities compared to bulk CoPc. Gas chromatograph (GC) analysis (Figure S9) identified CO and H_2_ as the sole gaseous products, with no liquid products detected in ^1^H nuclear magnetic resonance (NMR) analysis of electrolytes (Figure S10). Over the potential range of −0.8 to −1.1 V versus RHE, *FE*_CO_ remained above 90.1% for N‐CoPc/CNT‐COOH. Optimal performance was observed at −1.0 V versus RHE, where *FE*_CO_ reached 96.6%, and the partial current *j*_CO_ attained 18.3 mA cm^−^^2^. In contrast, bulk CoPc exhibited lower product selectivity, with a maximum *FE*_CO_ of only 81.4% within the same potential window. These results clearly demonstrate the superior catalytic activity of N‐CoPc/CNT‐COOH for CO_2_RR relative to bulk CoPc. Assuming Co‐N_4_ as catalytically active sites, we calculated the turnover frequency (TOF) by normalizing the *j*_CO_ over the number of these Co sites (Figure S11). The TOF of N‐CoPc/CNT‐COOH increased monotonically with applied potential, peaking at 69,580 h^−1^ at −1.0 V versus RHE, which is 3.59 times higher than that of bulk CoPc (19,392 h^−1^). This high value reflects the significantly enhanced intrinsic activity of the Co‐N_4_ sites within the nanocomposite. The performance of N‐CoPc/CNT‐COOH was further benchmarked against the state‐of‐the‐art electrocatalysts for CO_2_‐to‐CO conversion in neutral electrolytes (Table S1), confirming its superior performance in terms of combined *FE*_CO_ and *j*_CO_.

**FIGURE 3:**
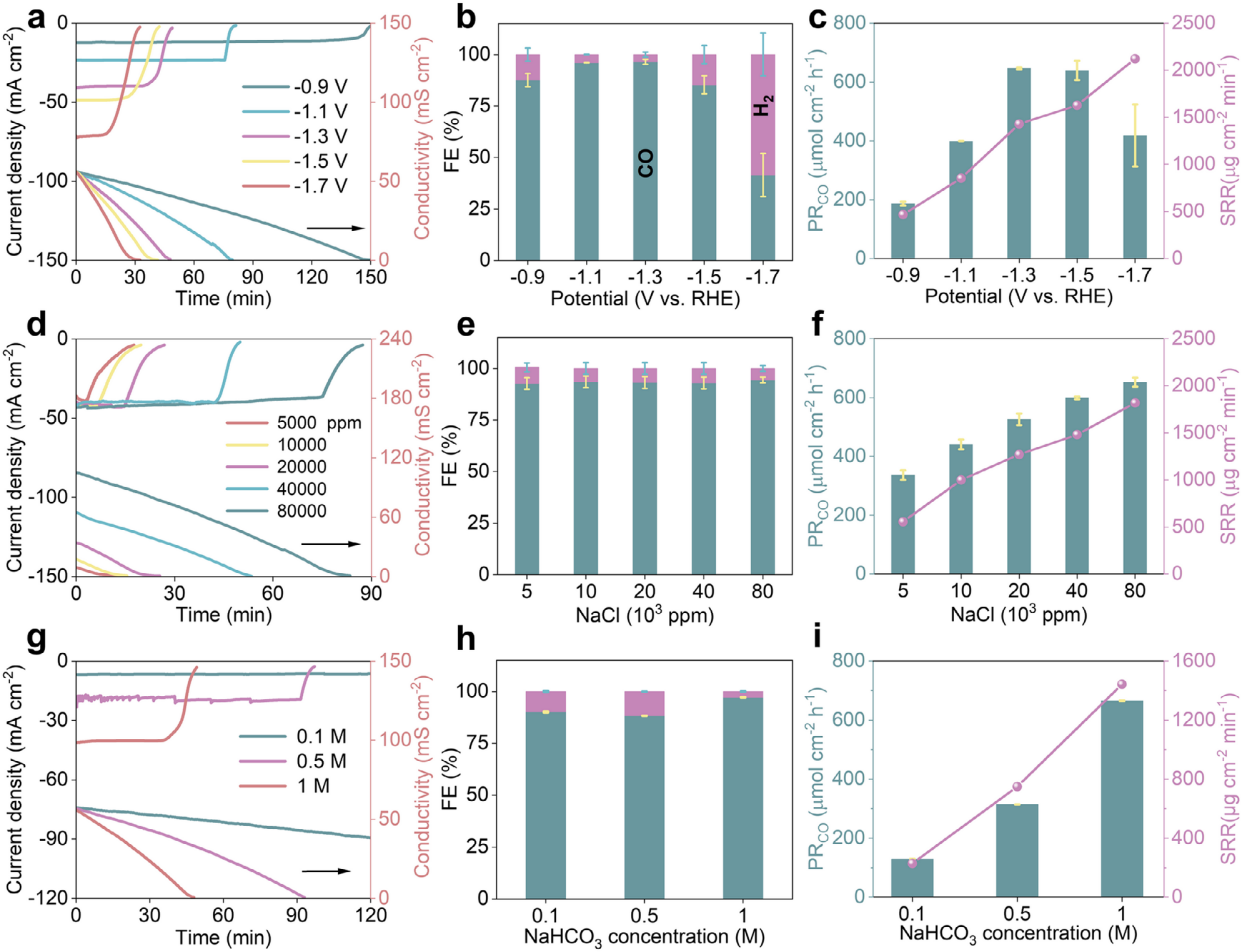
The performances of the electrocatalytic desalination device with a GDL coated with N‐CoPc/CNT‐COOH catalyst. (a) *I–t* curves and corresponding changes of ionic conductivity, (b) *FE*_CO_ and $FE_{H_{2}}$, (c) *PR*_CO_ and *SRR* delivered by the electrocatalytic desalination device at different potentials; (d) *I–t* curves and corresponding changes of ionic conductivity, (e) *FE*_CO_ and $FE_{H_{2}}$, (f) *PR*_CO_ and *SRR* delivered by the electrocatalytic desalination device processing brine with different NaCl concentrations from 5 to 80 ppm, (g) *I–t* curves and corresponding changes of ionic conductivity, (h) *FE*_CO_ and $FE_{H_{2}}$, (i) *PR*_CO_ and *SRR* delivered by the electrocatalytic desalination using NaHCO_3_ electrolytes at concentrations from 0.1 to 1 M.

Next, we integrated the electrocatalysts into the desalination device to compare their performance. To evaluate the influence of applied potentials on CO_2_RR and desalination efficiency, steady‐state current measurements were conducted over a range of −0.9 to −1.7 V versus RHE. Figure 3a shows that the ionic conductivity of 2 mL simulated seawater (∼55.2 mS cm^−^^2^, 35 g L^−^^1^ NaCl) reduced to below 1 mS cm^−^^2^ (corresponding to a drinking water level with 99% SRE after 29.5, 38.5, 48.5, 78.5, and 145.5 min under the applied potential of −1.7, −1.5, −1.3, −1.1, and −0.9 V vs. RHE, respectively). Higher applied potentials generated larger current densities, thereby accelerating the desalination process. N‐CoPc/CNT‐COOH maintained *FE*_CO_ above 85.3% across a broad potential window from −0.9 to −1.5 V versus RHE (Figure 3b), demonstrating its broad operating potential window. The highest *FE*_CO_ and *PR*_CO_ were achieved at −1.3 V versus RHE, yielding 96.5% and 646 µmol cm^−2^ h^−1^, respectively (Figure 3c), establishing this as the optimal potential. Under these conditions, the calculated *SRR* was 1422.7 µg cm^−2^ min^−1^. A comparative analysis with bulk CoPc (which exhibited 89.1% *FE*_CO_, 439 µmol cm^−2^ h^−1^ *PR*_CO_, and 972.0 µg cm^−2^ min^−1^ *SRR* at −1.3 V vs. RHE) clearly highlights the enhanced capability of N‐CoPc/CNT‐COOH to drive coupled seawater desalination and CO production (Figures S12 and S13). The improved desalination performance can be attributed to the high current density delivered by the efficient N‐CoPc/CNT‐COOH‐catalyzed CO_2_RR.

The CO_2_RR kinetics of N‐CoPc/CNT‐COOH were further probed by electrochemical impedance spectroscopy (EIS) and electrochemical active surface area (ECSA). Nyquist plots (Figure S14) and the fitted data (Table S2) indicate that N‐CoPc/CNT‐COOH has a lower interfacial charge transfer resistance (4.47 Ω) than bulk CoPc (8.93 Ω), suggesting the enhanced charge transport at the N‐CoPc/CNT‐COOH electrode's surface [51]. The ECSA was estimated from the electrochemical double‐layer capacitance (*C*_dl_) measured from CV curves in the non‐Faradaic region (Figure S15). Comparative analysis shows that N‐CoPc/CNT‐COOH (4.00 mF cm^−^^2^) exhibits a *C*_dl_ value 2.13 times larger than that of bulk CoPc (1.88 mF cm^−^^2^). Since ECSA is proportional to *C*_dl_, [52] this result indicates a substantially larger electroactive area and a higher density of accessible active sites in N‐CoPc/CNT‐COOH. Collectively, these findings confirm that both the nanorod structure of CoPc and its integration with CNT‐COOH are pivotal to achieving enhanced CO_2_RR performance.

Furthermore, we evaluated the effect of initial salt concentration on the electrocatalytic desalination efficiency. As shown in Figure 3d, at a fixed potential of −1.3 V versus RHE and varying salinity levels (5000–80,000 ppm NaCl), the current density remains stable across the range of initial salt concentrations. This stability is attributable to the solution's adequate conductivity even at 5000 ppm. A sharp decline in current density is observed in the final stage of desalination, due to reduced ionic conductivity as the salt is depleted. The time required to reduce conductivity to the freshwater benchmark (1 mS cm^−1^) was 10, 15.5, 26, 54, and 83.5 min at salinities of 5000, 8000, 10,000, 12,000, and 14,000 ppm, respectively. The device exhibits strong adaptability to different saline concentrations, with CO product selectivity consistently exceeding 92.0% (Figure 3e), enabling efficient desalination from brackish to highly concentrated brine. The corresponding *SRR* and *PR*_CO_ are presented in Figure 3f. The *SRR* increased with salt concentration, reaching a maximum of 1818.2 µg cm^−2^ min^−1^ at an initial salt concentration of 80,000 ppm. This enhancement arises because elevated salinity increases ionic conductivity, allowing the system to maintain a higher current density for a greater proportion of the desalination process, thereby accelerating the average *SRR* [53].

Finally, we optimized the circulating electrolyte concentration by fixing the potential at −1.3 V versus RHE and varying the NaHCO_3_ concentration (0.1, 0.5, and 1 M). Figure 3g demonstrates a positive correlation between the electrolyte concentration and the achieved current density. Desalination of the 35,000 ppm saline feed required 307, 93, and 48.5 min in 0.1, 0.5, and 1 M NaHCO_3_ electrolytes, respectively. The corresponding *SRR* values were 228.0, 748.7, and 1422.7 µg cm^−2^ min^−1^ in the 1 M NaHCO_3_ electrolyte, a performance attributed to increased ionic conductivity near saturation (1.14 M under standard conditions). N‐CoPc/CNT‐COOH maintained a *FE*_CO_ above 88.4% across all concentrations (Figure 3h), underscoring its robustness. The accumulation of HCO_3_^−^ at the electrode–electrolyte interface increases with electrolyte concentration, which is responsible for the elevated *FE*_CO_ in 1 M NaHCO_3_ electrolyte [35]. Combining parameters in *FE*_CO_, *SRR*, and *PR*_CO_ (Figure 3i), optimal performance was observed at 1 M, which was therefore selected as the optimal electrolyte concentration.

To further elucidate the kinetic influence on desalination efficiency, a linear regression analysis was conducted to correlate the *SRR* with *j*. As depicted in Figure S16, a robust linear relationship was observed, indicating that the electrochemical reactions (CO_2_RR/OER) dictate desalination kinetics by modulating the electron flux, which drives compensatory ion transport and thereby dominates salt removal. This establishes that the desalination rate is intrinsically coupled to the electrode reactions, with the applied current density acting as the fundamental driver of ion migration.

### 2.4 Long‐Term Performance of the Desalination Cell

**FIGURE 4:**
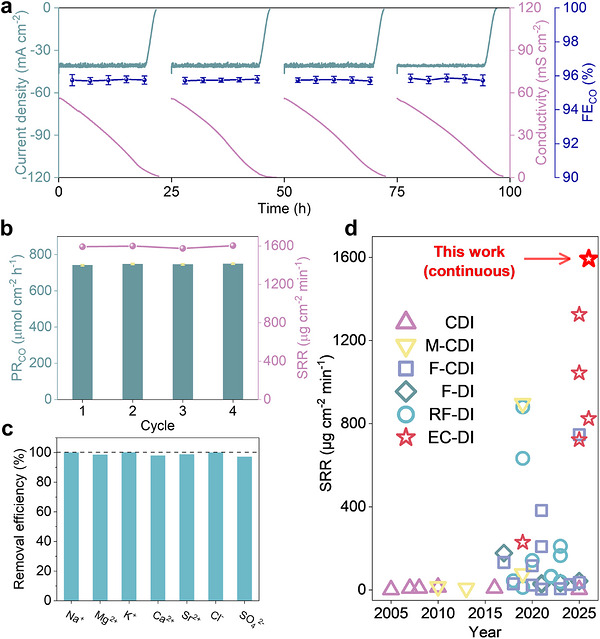
(a) The *I–t* curves and corresponding ionic conductivity changes of desalinated seawater and *FE*_CO_, (b) *PR*_CO_ and *SRR* delivered by the electrocatalytic desalination cell during long‐term stability test without refreshing its electrolyte (1 M NaHCO_3_). (c) Removal efficiency of diverse ions in natural seawater. (d) The *SRR* comparison of different desalination techniques (the data are obtained from Table S5).

The long‐term stability of the desalination cell was evaluated using a large‐volume, multi‐cycle natural seawater desalination protocol at its optimal operation conditions (–1.3 V vs. RHE, 1 M NaHCO_3_ electrolyte). Each cycle processed 60 mL of natural seawater; four consecutive cycles were performed without refreshing the circulating electrolyte. The desalination duration per cycle remained stable at approximately 22 h, resulting in a total operational time of nearly 90 h (Figure 4a). Throughout this extended process, the cell demonstrated exceptional stability and robustness. The current density was consistently maintained at ∼40 mA cm^−2^, while *FE*_CO_ of the N‐CoPc/CNT‐COOH cathode remained between 95.5% and 96.4%, with $FE_{H_{2}}$ suppressed below 5% (Figure S17). Furthermore, as shown in Figure 4b, the device exhibited remarkable production stability, with the average *PR*_CO_ per cycle remained above 741.5 µmol cm^−2^ h^−1^ equal to 3986 L day^−1^ m^−2^, and the average *SRR* per cycle ranged from 1575.0 to 1603.6 µg cm^−2^ min^−1^ equal to freshwater production rate (PR_freshwater_) of 642.9.0 to 654.6 L day^−1^ m^−2^ (Figure S18). Additionally, a synchronous trend was observed between the current density and the conductivity derivative (Figure S19), where the desalination rate increased gradually during the initial phase (∼17 h) before declining. This behavior is primarily attributed to the diminishing salt concentration in the feed solution during the final stages of desalination.

The ionic composition of the desalinated natural seawater and circulating electrolyte was analyzed using inductively coupled plasma optical emission spectroscopy (ICP‐OES) and ionic chromatography (IC). Figure 4c and Table S3 confirm that the total SRE exceeds 99% across various ions, meeting potable water standards. Table S4 and Figure S20 show that the concentrations of Ca^2+^, Mg^2+^, and Cl^–^ ions remained very low, with only minimal variation. No precipitation or the formation of corrosive chlorine species in the electrolyte chamber were observed. To assess catalyst durability, post‐reaction analysis of N‐CoPc/CNT‐COOH was conducted. LSV curves (Figure S21a) showed minimal performance degradation. Subsequent structural characterization using TEM, Raman spectroscopy, and XPS (Figure S21b–d) revealed that the catalyst retained its structural and compositional integrity, demonstrating excellent durability under the applied operational conditions.

The average *SRR* calculated across this long‐term stability test was 1592.8 µg cm^−2^ min^−1^. As summarized in Table S5 and illustrated in Figure 4d, this value represents one of the highest *SRRs* reported to date, significantly surpassing those of other desalination techniques, such as capacitive deionization (CDI) [54], Faradaic deionization (FCDI) [55], and redox flow desalination (RFD) [56]. Furthermore, the *SRR* reported in this work exceeds previously reported values for electrocatalytic desalination [13, 31].

From an energy perspective, the specific energy consumption of this device for natural seawater desalination is calculated to be 8.57 kWh m^−^^3^, comparable to advanced electrical methods such as RO, as shown in Table S6. The overall energy efficiency (*EE*_total_) of this device is calculated to be 16.72% (see calculation details in Supporting Information), which is within the normal range for CO_2_RR/OER systems in a flow‐cell configuration with a neutral electrolyte [35, 57]. Notably, it achieves an unprecedented *SRR* of 1592.8 µg cm^−^^2^ min^−^^1^ via a single‐step, nonpressure‐driven process that enables >99% desalination.

### 2.5 TEA of the Desalination Cell

We evaluated the economic viability of the desalination cell with 1 m^2^ cross‐sectional area through a comprehensive TEA, analyzing the full spectrum of operational expenditures, including electrolytes, IEMs, electricity, and so on (Tables S7–S10, see details in the Supporting Information). The comparative data in Table S7 demonstrate that although our device incurs a slightly higher membrane replacement cost, the implemented electrolyte circulation strategy significantly reduces daily electrolyte cost to $0.185/day, outperforming existing literature values ranging from $6.19/day to $35.20/day [19, 31, 32]. This performance surpasses prior benchmarks and underscores the device's exceptional efficiency and practical viability for sustainable seawater treatment.

To evaluate real‐world applicability, we performed region‐specific economic assessments using local water and electricity pricing structures. The analysis reveals high sensitivity to electricity costs, and co‐produced freshwater revenue effectively subsidizes electrical expenditures. In the Egyptian case study, the net production cost of CO (after deducting freshwater revenue) is $639/ton_CO_, demonstrating strong competitiveness against the international CO production price of approximately $600/ton_CO_. Under ideal electricity pricing conditions of $0.03/kWh (benchmark for CO_2_RR), and seawater desalination cost of $6.36/ton_freshwater_ using RO, the net production cost reaches –$63/ton_CO_, confirming that freshwater production revenue completely offsets the CO_2_RR costs and highlighting the significant economic advantage of our integrated system.

## 3 Conclusion

In conclusion, this work resolves long‐standing barriers in electrocatalytic desalination by simultaneously achieving continuous operation, ultrafast desalination kinetics, and dramatically reduced operating costs. Through the rational integration of a high‐performance N‐CoPc/CNT‐COOH electrocatalyst with a uniquely designed five‐chamber reactor, we establish a self‐balanced circulating electrolyte system that eliminates the need for electrolyte replacement—a critical limitation in prior designs. This architecture not only suppresses electrolyte degradation and byproduct accumulation but also sustains high current densities required for rapid ion transport. As a result, the system delivers a record‐high *SRR* of 1592.8 µg cm^−^^2^ min^−^^1^ during ∼90 h of stable operation using natural seawater, while maintaining >95.5% Faradaic efficiency for CO production and producing potable‐quality freshwater (>99% salt removal). Importantly, TEA reveals that the electrolyte‐free operating mode reduces daily electrolyte costs by more than an order of magnitude compared with previous electrocatalytic desalination systems. By unifying continuous desalination, CO_2_ valorization, and cost‐effective operation within a single electrochemical platform, this study provides a viable blueprint for scalable, sustainable seawater treatment technologies and advances the practical deployment of electrochemical water–carbon nexus systems.

## Author Contributions

**Man Liang**: methodology, data curation, formal analysis, writing – original draft. **Pucheng Duan**: formal analysis, methodology. **Minzhang Li**: conceptualization, methodology, data curation, formal analysis, writing – original draft, funding acquisition. **Zhefei Wu**: formal analysis, investigation, methodology. **Lu Guo**: formal analysis, writing – original draft, data curation. **Afzalshoh Qahramon Zarifzoda**: formal analysis, methodology, investigation. **Chengli Rong**: formal analysis, writing – review and editing. **Fuming Chen**: conceptualization, writing – original draft, funding acquisition, supervision, project administration. **Yuan Chen**: funding acquisition, writing – review and editing, conceptualization.

## Conflicts of Interest

The authors declare no conflicts of interest.

## Supporting information




**Supporting File**: anie72445‐sup‐0001‐SuppMat.docx.

## Data Availability

The data that support the findings of this study are available from the corresponding author upon reasonable request.
