# Supplementary material for: Electrolyte‐Replacement‐Free Continuous Electrocatalytic Desalination Coupled With CO2 Reduction at Record Throughput and Low Cost
Source: Angew Chem Int Ed Engl. 2026 May 1;65(25):e9124699. doi: 10.1002/anie.9124699 (PMC13266946; doi:10.1002/anie.9124699)
Supplement: Supplementary file 1 — Supporting File: anie72445‐sup‐0001‐SuppMat.docx. [file ANIE-65-e9124699-s001.docx]

**Supporting Information**

**Electrolyte-Replacement-Free Continuous Electrocatalytic Desalination Coupled with CO₂ Reduction at Record Throughput and Low Cost**

Man Liang^1,2#^, Pucheng Duan^2#^, Minzhang Li^2^*, Zhefei Wu^2^, Lu Guo^3^, Afzalshoh Qahramon Zarifzoda^4^, Chengli Rong^5^, Fuming Chen^1,2,3^*, Yuan Chen^5*^

*^1^ State Key Laboratory of Tropic Ocean Engineering Materials and Materials Evaluation, School of Chemistry and Chemical Engineering, Hainan University, Haikou, 570228 China*

*^2^ Guangdong Provincial Key Laboratory of Quantum Engineering and Quantum Materials, Guangdong Engineering Technology Research Center of Efficient Green Energy and Environment Protection Materials, School of Electronic Science and Engineering (School of Microelectronics), South China Normal University, Foshan 528225, China*

*^3^ Yunnan-Malaya Institute, School of Engineering, Yunnan University, Kunming 650091, China*

*^4^ S.U.Umarov Physical-Technical Institute of the National Academy of Sciences of Tajikistan, Dushanbe, 734025, Tajikistan*

*^5^ School of Chemical and Biomolecular Engineering, The University of Sydney, Darlington, New South Wales, 2006, Australia*

*# These authors contributed equally.*

***Email****:* [liminzhang@scnu.edu.cn](mailto:liminzhang@scnu.edu.cn) *(M. Li);* [fmchen@hainanu.edu.cn](mailto:fmchen@m.scnu.edu.cn) *(F. Chen);* [*yuan.chen@sydney.edu.au*](mailto:yuan.chen@sydney.edu.au) *(Y. Chen)*

***Chemicals and materials***

All reagents and chemicals were commercially obtained and used as received. Cobalt(II) phthalocyanine (CoPc, ≥95%), methanesulfonic acid (CH_4_O_3_S, ≥98%), ethanol (C_2_H₅OH, ≥99.7%), isopropyl alcohol (C_3_H_8_O, ≥99.7%), sodium bicarbonate (NaHCO_3_, ≥99.95%), and Nafion solution (D521CS, 5.0–5.4% polymer content) were purchased from Aladdin. Carboxylated multi-walled carbon nanotubes (CNT-COOH, XFM03) were supplied by Nanjing XFNANO Materials Tech. Carbon cloth (WOS1002, thickness 0.3 mm) was acquired from CeTech. Sigracet 36BB gas diffusion layers (GDL) with a dual-layer structure, consisting of 5 wt% PTFE-treated carbon paper and a teflonized microporous layer, were obtained from SCI Materials Hub. Deionized (DI) water (electrical resistivity >18.2 MΩ cm⁻^1^) was produced using a Millipore Milli-Q system and employed in all experiments. Ultrahigh-purity CO_2_ (99.999%) and Ar (99.999%) were provided by Guangzhou Yuejia Gas Co., Ltd. Anion exchange membranes (ASE) and cation exchange membranes (CSE) were procured from ASTOM Corporation, Japan.

***Synthesis of N-CoPc/CNT-COOH electrocatalyst***

N-CoPc/CNT-COOH was synthesized by a precipitation method, as illustrated in Figure S4. Briefly, 20 mg of CNT-COOH powder was dispersed in 80 mL of CH_4_O_3_S, and the mixture was sonicated for over 1 h to obtain a uniformly dispersed suspension. Subsequently, 40 mg of CoPc powder was introduced into the suspension, followed by an additional 30 min of sonication to ensure complete molecular-level dissolution of CoPc. The resulting mixture was then dripped batchwise (5 mL per batch) into 200 mL of rapidly stirred DI water. After completion of the dripping process, the mixture was filtered, and the solid product was thoroughly washed with ethanol and DI water until the filtrate reached a neutral pH. Finally, N-CoPc/CNT-COOH was dried under vacuum at 80 °C.

***Catalyst characterization***

Transmission electron microscopy (TEM) and high-resolution TEM (HR-TEM) imaging were collected on a JEOL JEM-2100F microscope operating at 200 kV. Elemental mapping via energy-dispersive X-ray spectroscopy (EDS) was carried out using an Oxford Instruments detector attached to the TEM. Fourier-transform infrared (FTIR) spectra were recorded in the range of 400–4000 cm⁻^1^ using a Bruker Tensor 27 spectrometer in KBr pellets. Raman spectra were acquired on a Horiba LabRAM HR Evolution spectrometer equipped with a 532 nm laser excitation source. Ultraviolet-visible diffuse reflectance spectroscopy (UV-Vis DRS) measurements were performed on a Shimadzu UV-2600 spectrophotometer, analyzing ethanol-dispersed catalysts over the 400–800 nm wavelength range. X-ray diffraction (XRD) patterns were obtained using a Bruker D8 Advance diffractometer with Cu Kα radiation (λ = 1.5418 Å). Surface chemical states were examined by X-ray photoelectron spectroscopy (XPS) using a Thermo Scientific K-Alpha+ system with a monochromatic Al Kα source, and all XPS spectra were energy-calibrated by referencing the C 1*s* peak to 284.8 eV.

***Fabrication of working electrodes***

The electrocatalyst ink was formulated by combining 1.2 mL of isopropanol, 0.7 mL of DI water, 100 μL of 5 wt% Nafion solution, 8 mg of catalyst powder, and 2 mg of Ketjenblack, followed by 60 min of ultrasonication at 40 kHz to attain a colloidally homogeneous dispersion. This ink was then uniformly sprayed onto a 1 × 1 cm^2^ region of a gas diffusion layer (GDL) to achieve a precise catalyst mass loading of 0.8 mg cm⁻^2^, after which the electrodes were vacuum-dried for 12 h to ensure thorough solvent removal and foster robust catalyst-carbon interfacial adhesion.

***CO_2_RR performance measurements***

The performance of working electrodes for the electrocatalytic CO_2_ reduction reaction (CO_2_RR) was evaluated under ambient conditions using a flow cell (Gaoss Union Photoelectric Technology Co., Ltd., Tianjin) constructed using acrylic materials. The cell consisted of cathode and anode chambers with identical thicknesses of 1 cm and cross-sectional areas of 1 × 1 cm^2^, separated by an anion exchange membrane (AEM). A catalyst-coated GDL with an area of 1 × 1 cm^2^, fabricated as described above) served as the working electrode in the cathode chamber, paired with an Ag/AgCl (saturated KCl) reference electrode, while a commercial IrO_2_/Ti electrode (Gaoss Union Photoelectric Technology Co., Ltd., Tianjin) was employed for the oxygen evolution reaction (OER) in the anode chamber, with both chambers filled with 1.0 M NaHCO_3_ electrolyte. All electrochemical measurements were conducted using a CHI760E workstation (Shanghai Chenhua Instrument Co., Ltd.), including linear sweep voltammetry (LSV) at 20 mV s⁻^1^, electrochemical impedance spectroscopy (EIS) from 10 kHz to 0.05 Hz with a 20 mV bias, electrochemical active surface area (ECSA) determination via cyclic voltammetry (CV) at scan rates of 20–100 mV s⁻^1^ in a non-Faradaic window, turnover frequency (TOF) assessment by CV at 20 mV s⁻^1^ in the Faradaic region, and double-layer capacitance (C_dl_) calculation from capacitive current–scan rate regression. All potentials were converted to the reversible hydrogen electrode (RHE) scale using E_RHE_ = E_Ag/AgCl_ + 0.197 + 0.059 × pH without iR compensation.

***CO_2_RR product analysis***

Gaseous products were quantified using a gas chromatograph (GC, Fuli 7900II) equipped with a flame ionization detector (FID) and a thermal detector (TCD). High-purity argon (99.999%) served as the carrier gas. The outlet gas flow rate was monitored in real time with a digital flow meter (Siargo Ltd., MF4701). The GC was calibrated prior to measurements using certified standard gases. Each gas-phase sample was analyzed for 15 min, and all tests were repeated at least twice. Error bars represent the relative deviation between replicates.

The faradaic efficiency of gaseous products (H_2_ and CO) was calculated as follows.

$$FE\left( \% \right)=\frac{(\frac{V}{60s {min}^{-1})}\times\left( \frac{y}{24000 cm^{3} mol^{-1}} \right)\times N\times F}{I}\times100\%$$

where *V* is the CO_2_ flow rate (mL min^−1^), *y* is the product concentration (vol%) determined by GC calibration, *N* is the number of electrons transferred (*N* = 2 for CO or H_2_), *F* is the Faraday constant (96485 C mol^−1^), and *I* is the total current (A).

The formation rate of CO (*R_CO_*) can be calculated as follows:

$$R_{CO}=\frac{Q_{total}\times1000}{F\times N\times t\times S}\times FE_{CO}$$

where *Q_total_* is the total charge (C), *N* is the number of electrons transferred from CO_2_ to CO, t is the reaction time, and *S* is the effective surface area of the working electrode.

Liquid products were analyzed by ^1^H NMR spectroscopy on a Bruker AVANCE NEO 600 MHz spectrometer, using dimethyl sulfoxide (DMSO) as an internal standard. For analysis, 500 μL of the electrolyte was mixed with 70 μL of D_2_O and 300 μL of a 10 mM DMSO solution. ^1^H NMR spectra were acquired using the presaturation method to suppress the water signal.

***Electrochemical CO_2_RR-driven seawater desalination***

The electrocatalytic CO_2_RR-driven seawater desalination system was constructed by integrating two seawater chambers between the cathode and anode compartments of a conventional CO_2_RR flow cell. The cathode and anode chambers were interconnected to form a shared electrolyte chamber, which was separated from the two seawater chambers by cation-exchange membranes (CEM), while the two seawater chambers were divided by an AEM. The assembly was configured in the following order: CO_2_ gas chamber | GDL (working electrode) | electrolyte chamber | CEM | desalination chamber | AEM | concentration chamber | CEM | electrolyte chamber | counter electrode.

The seawater chambers were fabricated using silicone plates (thickness: 0.3 cm; cross-sectional area: 1×1 cm^2^). The working electrode preparation, reference electrode (Ag/AgCl, saturated KCl), counter electrode (IrO_2_/Ti), and electrolyte system remained consistent with the standard CO_2_RR flow cell configuration. The geometric areas of the ion-exchange membranes (CEM and AEM) and the electrodes (cathode and anode) were precisely matched to the cross-sectional area of the silicone plates (1 cm^2^) to ensure uniform current distribution and consistent active areas throughout the cell. The central saline chamber was filled with either simulated seawater (35 g L⁻^1^ NaCl in DI water, initial conductivity ~55.2 mS cm⁻^1^) or natural seawater collected from the South China Sea (initial conductivity ~56.0 mS cm⁻^1^, salinity ~35,000 ppm).

Constant-potential electrolysis was conducted at various potentials (−0.9 to −1.7 V vs. RHE). Real-time monitoring of conductivity changes in the central chamber was performed using a conductivity meter (eDAQ EPU357 with ET908 probe). The pH of the solution was measured with a laboratory bench-top pH meter (METTLER TOLEDO FiveEasy Plus FE28, equipped with LE438-2M IP67 electrode probe), with readings taken at 5-minute intervals.

To assess the potential generation of corrosive chlorine species (e.g., Cl_2_, HOCl, OCl⁻) in the circulating electrolyte, the concentration of free chlorine was quantified using the DPD (N,N-diethyl-p-phenylenediamine) spectrophotometric method. Briefly, a commercial DPD reagent sachet (Thermo Orion AC4P72) was added to a vial containing the electrolyte sample, followed by vigorous shaking for approximately 10 s. In the presence of free chlorine, the solution develops a pink coloration proportional to the concentration. The reacted sample was immediately transferred to a quartz cuvette within 2 min, and the absorbance was measured using a UV-Vis spectrophotometer in dual-beam mode. The characteristic absorbance peak for the reaction product between DPD and free chlorine species (primarily OCl⁻ under alkaline conditions) was observed at 552 nm. To validate the detection capability, a NaClO standard solution (1 mg L⁻^1^) was measured as a positive control, exhibiting a distinct absorbance peak at 552 nm, while the electrolyte samples before and after operation showed negligible absorbance at this wavelength, indicating the absence of detectable free chlorine.

***Calculations of SRE and SRR***

The salt removal efficiency (SRE) and the average salt removal rate (SRR, *μ*g cm⁻^2^ min⁻^1^) were calculated using:

$$SRE=\frac{C_{0}-C_{t}}{C_{0}}\times100\%$$

$$SRR=\frac{(C_{0}-C_{t})\times V}{S\times t}$$

where $C_{0}$ (mg L⁻^1^) and $C_{t}$ (mg L⁻^1^) are the salt concentrations at the initial;$V$ represents the volume of the salt solution (mL); $S$ is the electrodes’ active area (cm^2^), and $t$ (min) is the time for desalination.

***Calculations of EC_desalination_***

Specific energy consumption for desalination (EC_desalination_) was calculated as:

$$EC_{desalination}=\frac{\Delta E}{V}=\frac{{(U}_{integrated} -U_{standalone}) \times Q}{V}$$

$$=\frac{\left( 5.42 V-4.86 V \right) \times110.2 C}{2\times{10}^{-6}m^{3}} =30856 kJ/m^{3}\approx8.57 kWh/m^{3}$$

where *U_integrated_* and *U_standalone_* represent the full-cell voltages of the electrocatalytic desalination device coupled with seawater chambers and the CO_2_RR flow cell device without seawater chambers, respectively, under optimal seawater desalination conditions (at a current density of ~41 mA cm⁻^2^); *V* denotes the volume of desalinated seawater; and *Q* represents the total charge.

***Calculations of EE_total_***

The overall energy efficiency is calculated as follows:

$$EE_{total}=\frac{TSEC_{desalination}\times V+TSEC_{CO2RR}\times n_{CO}+TSEC_{OER}\times n_{O2}}{EC_{total}}=\frac{TSEC_{desalination}\times V}{EC_{total}}+\frac{TSEC_{CO2RR}\times n_{CO}+TSEC_{OER}\times n_{O2}}{EC_{total}}=\left[ \frac{-d\left( \Delta G_{mix} \right)\times V}{U_{integrated}\times Q}+\frac{E_{O2}{-E}_{CO}}{U_{integrated}}\times{FE}_{CO} \right]\times100\%=\left( \frac{-R\times T\times V\times lna_{w}dn_{w}}{U_{integrated}\times Q}+\frac{E_{O2}{-E}_{CO}}{U_{integrated}}\times{FE}_{CO} \right)\times100\%=\left( \frac{1.06 kWh/m^{3}\times3\times{10}^{-6} m^{3}}{5.42 V\times110.2 C}+\frac{0.74 V+0.1 V}{5.42 V}\times95.5 \% \right)\times100\%=\left( 0.01917+0.14801 \right)\times100\%=16.72\%$$

Where EE_total_ represents the overall energy efficiency of the electrocatalytic desalination device (%). TSEC_i_ denotes the theoretical specific energy consumption for each corresponding process: TSEC_desalination_ represents the theoretical specific energy required per cubic meter of freshwater produced (kWh/m^3^); TSEC_CO2RR_ and TSEC_OER_ represent the theoretical specific energy consumption per mole of product (kWh/mol, CO and O_2_, respectively). n_CO_ and n_O2_ denote the number of moles of CO and O_2_ produced (mol), respectively. EC_total_ is the total electrical energy input to the system (kWh).

Specifically, the TSEC_desalination_ is derived from the Gibbs free energy of mixing (ΔG_mix_), where ΔG_mix_ is the free energy of mixing, R is the ideal gas constant, T is the absolute temperature, a_w_ is the activity of water, n_w_ is the number of moles of water. When the volume ratio of desalinated seawater to concentrated seawater is 1:1, the minimum theoretical specific energy consumption (-RT lna_w_dn_w_) equals 1.06 kWh/m^3^_._^[123]^ In our calculation, V denotes the volume of desalinated seawater (m^3^, derived from a 3 mL feed volume), and Q represents the total charge passed during the desalination of this 3 mL feed (C). The energy efficiency for the electrochemical components is calculated based on the ratio of the theoretical potentials to the actual applied voltage.^[124]^ E_O2_ and E_CO_ are the standard potentials for the corresponding reactions, where E_CO_ is the standard potential for CO production (-0.1 V vs. RHE),^[125]^ FE_CO_ is the Faradaic efficiency of CO, and U_integrated_ is the applied cell voltage.

***Techno-Economic Analysis of the Desalination Cell***

The techno-economic analysis is based on an electrocatalytic desalination device with an 1 m^2^ cross-sectional area. The daily production rates (*DP*) of both freshwater and CO (ton/day) can be derived from the production rate (*PR*) through unit conversion：

$${DP}_{CO}={PR}_{CO}=741.5 \mu mol/({cm}^{2} h)\times28 g/mol\times1 m^{2}=0.0050 ton_{CO}/day$$

$${DP}_{freshwater}={PR}_{freshwater}=642.9 L/(m^{2} day)\times0.001 ton/L\times1 m^{2}=0.6429 ton_{freshwater}/day$$

The ratio:

$$\frac{{DP}_{CO}}{{DP}_{freshwater}}=\frac{0.0050 ton_{CO}/day}{0.6429 ton_{freshwater}/day}=\frac{1 ton_{CO}}{128.6 ton_{freshwater}}$$

As demonstrated in the calculation above, the co-production ratio indicates that for every 1 ton of CO produced, 128.6 tons of seawater are simultaneously desalinated into freshwater. Given this coupled production relationship, the total production cost of the electrocatalytic desalination device is expressed in terms of $/ton_CO_, with CO production serving as the calculation basis.

The total production cost is calculated as the sum of individual cost components associated with the operation and maintenance of the system:

$${PC}_{total}={PC}_{electrolyte}+{PC}_{membrane}+{PC}_{device}+{PC}_{cathode}+{PC}_{electricity}+{PC}_{separation}+{PC}_{CO2}{+PC}_{other}$$

where:

*PC_i_* denotes the specific production cost ($/ton_CO_) attributed to each component I;

*PC_electrolyte_* and *PC_membrane_* account for the costs of electrolyte and membrane replacement, respectively;

*PC_device_* refers to the amortized capital cost of the device infrastructure;

*PC_cathode_* represents the cost associated with cathode catalyst replacement;

*PC_electricity_* covers the cost of electrical energy consumption during operation;

*PC_separation_* includes the costs for CO product separation and purification;

*PC_CO2_* denotes the cost of the CO_2_ feedstock;

*PC_other_* encompasses other miscellaneous operational costs.

This comprehensive breakdown ensures that all critical economic factors are accounted for in the techno-economic analysis (Cost Basis & Assumptions: exchange rate of 1 USD = 6.99 CNY)

Specifically, the replacement costs for the ion-exchange membrane and electrolyte are calculated based on the daily average operating costs derived from **Table S7**. The daily cost is calculated using the following equation:

$$C_{electrolyte}=\sum m_{i}\times p_{i}=V_{electrolyte}\times\left( \sum c_{i}{\times M}_{i}\times p_{i} \right)$$

$$C_{membrane}=\left（ n\times S\times p_{m} \right） / T_{m}$$

where:

*C_electrolyte_*: Daily cost of electrolyte replacement ($/day).

*m_i_*: Daily consumption of the i-th solute (kg/day).

*p_i_*: Market price of the i-th solute ($/kg).

*V_electrolyte_*: Daily volume of electrolyte replaced (m^3^/day).

*c_i_*: Molar concentration of the i-th solute (mol/m^3^).

*M_i_*: Molar mass of the i-th solute (kg/mol).

*C_membrane_*: Daily cost of membrane amortization ($/day).

*n*: Number of membranes used in the device.

*S*: Cross-sectional area of the device (m^2^).

*p_m_*: Unit price of the membrane ($/m^2^).

*T_m_*: Service life of the membrane (year).

Replacement frequency assumptions: The electrolyte replacement frequency significantly impacts the total operational cost. For conventional three-chamber electrocatalytic desalination devices (three membranes) reported in the literature, the electrolyte is assumed to need complete replacement after each desalination cycle to maintain performance. In contrast, for the device designed in this work(four chambers with three membranes), the electrolyte in the catalytic chamber remains chemically stable over extended operation; therefore, its replacement frequency is assumed to be monthly (once every 30 days) for this economic analysis.

Subsequently, the production costs for the ion-exchange membrane and electrolyte is determined as follows:

$${PC}_{membrane}=\frac{C_{Membrane}}{{DP}_{CO}}=\frac{0.169 \$/day}{0.0050 ton_{CO}/day}=33.8 \$/\mathrm{ton}_{\mathrm{CO}}$$

$${PC}_{electrolyte}=\frac{C_{Electrolyte}}{{DP}_{CO}}=\frac{0.013 \$/day}{0.0050 ton_{CO}/day}=2.6 \$/\mathrm{ton}_{\mathrm{CO}}$$

Similarly, the device-related production cost is calculated using the daily average operating cost:

$${PC}_{device}=\frac{C_{device}}{{DP}_{CO}}=\frac{0.3855 \$/day}{0.0050 ton_{CO}/day}=77.1 \$/\mathrm{ton}_{\mathrm{CO}}$$

The detailed breakdown of material costs and the calculated daily average costs (amortized over a 15-year service life) are provided in **Table S8**.

The cathode production cost is calculated based on the daily average operating cost derived from **Table S9**:

$${PC}_{Cathode}=\frac{C_{cathode}}{{DP}_{CO}}=\frac{1.1708 \$/day}{0.0050 ton_{CO}/day}=234.1 \$/\mathrm{ton}_{CO}$$

The CO product separation cost is estimated based on literature values from reference,^[126]^ which employed pressure swing adsorption (PSA) technology for separation from industrial biogas streams, combined with Sherwood diagram analysis for cost scaling:

$${PC}_{separation}=10 \$/\mathrm{ton}_{\mathrm{CO}}$$

The CO_2_ feedstock gas cost and other operational costs are referenced from similar ion-exchange membrane-based flow cell electrocatalytic CO_2_-to-CO production systems reported in literature,^[127]^ with the following values:

$${PC}_{CO2}=0.063 \$/kg=63 \$/\mathrm{ton}_{\mathrm{CO}}$$

$${PC}_{other}=0.022 \$/kg=22 \$/\mathrm{ton}_{\mathrm{CO}}$$

To evaluate the global applicability and economic viability of the proposed system, we conducted a comparative economic analysis across various regions with distinct electricity tariffs and water costs (Table S10). A detailed case calculation using Egypt as an example is provided below to illustrate the cost breakdown methodology.

$${PC}_{electricity}=\frac{TSEC_{desalination}\times V+TSEC_{CO2RR}\times n_{CO}+TSEC_{OER}\times n_{O2}}{EE_{total}{\times DP}_{CO}}\times p_{ec}=\frac{EC_{total}}{{DP}_{CO}}\times p_{ec}=\frac{U_{integrated}\times j\times S}{{DP}_{CO}}\times p_{ec}=\frac{5.42 V\times40 mA/{cm}^{2}\times1 m^{2}\times24 h/day}{0.0050 ton_{CO}/day}\times0.021 \$/kWh=218.5 \$/ton_{CO}$$

Where *PC_electricity_* represents the electricity component of the production cost. *EE_total_* represents the overall energy efficiency of the electrocatalytic desalination device (%). *TSEC_i_* denotes the theoretical specific energy consumption for each corresponding process: *TSEC_desalination_* represents the theoretical specific energy required per cubic meter of freshwater produced (kWh/m^3^); *TSEC_CO2RR_* and *TSEC_OER_* represent the theoretical specific energy consumption per mole of product (kWh/mol, CO and O_2_, respectively). n_CO_ and n_O2_ denote the number of moles of CO and O_2_ produced (mol), respectively. *EC_total_* is the total electrical energy input to the system (kWh). *DP_CO_* is the daily CO production. p_ec_ is the electricity price. *j* is the current density under optimal operating conditions. *S* is the device area, taken as 1 m^2^ for amplification. *U_integrated_* represents the full cell voltage of the integrated electrocatalytic desalination coupling device under optimal operating conditions.

The total CO production cost is calculated by summing all component costs:

$${PC}_{total}={PC}_{electrolyte}+{PC}_{membrane}+{PC}_{device}+{PC}_{cathode}+{PC}_{electricity}+{PC}_{separation}+{PC}_{CO2}{+PC}_{other}=(33.8+2.6+77.1+234.1+218.5+10+63+22) \$/ton_{CO}\approx661 \$/ton_{CO}$$

The potential profit from desalination is calculated as:

$${PF}_{desalin}=p_{wa}\times\frac{{DP}_{freshwater}}{{DP}_{CO}}=0.17 \$/ton_{freshwater}\times128.6 ton_{freshwater}/ton_{CO}\approx22 \$/ton_{CO}$$

Where PF_desalin_ represents the potential profit from desalination. p_wa_ represents the local fresh water price for the corresponding region. DP_freshwater_ is the daily freshwater production. DP_CO_ is the daily CO production. Thus, the net CO production cost after accounting for desalination benefits is:

$$\mathrm{NC}_{\mathrm{CO}}={PC}_{total}-{PF}_{desalin}=(661-22) \$/ton_{CO}=639 \$/ton_{CO}$$

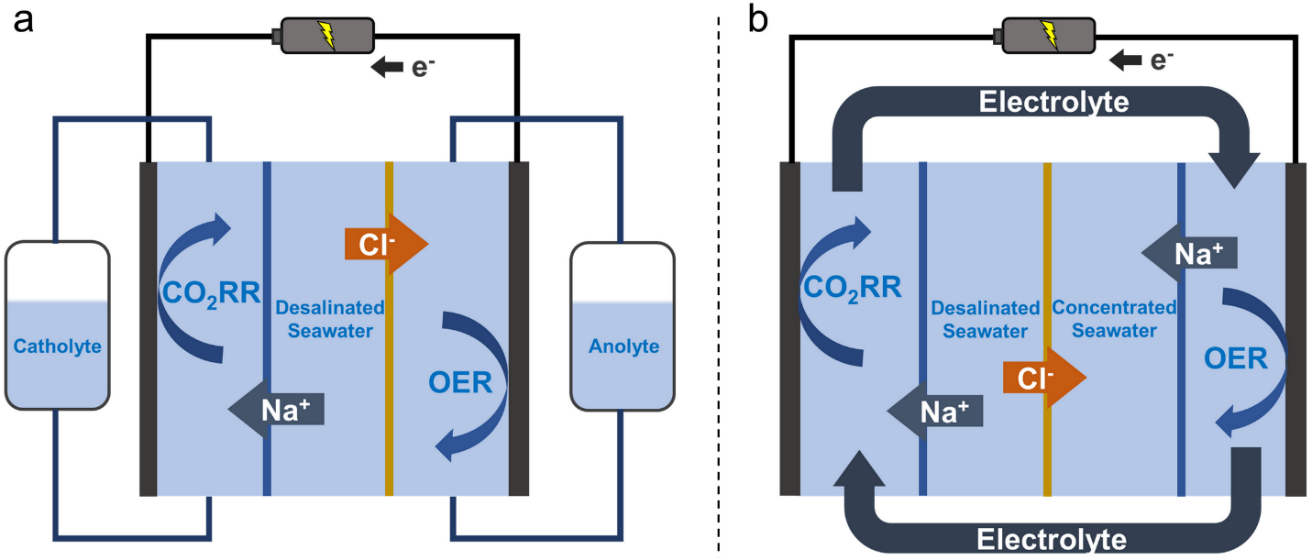


**Figure S1.** Schematic diagrams of a) a previously developed electrocatalytic desalination device and b) the new electrocatalytic desalination device reported in this work, with four electrolyte chambers and electrolyte circulation to enable continuous operation.

**Figure S2.** Time-dependent pH variations in individual chambers during electrocatalytic desalination of natural seawater. The pH divergence at the end of operation is attributed to competitive anion transport through AEM and subsequent OH^-^ migration (water naturally dissociates in the desalination chamber, with concentrations approximately 3×10^-6^ to 3×10^-7^ mol/L at pH = 7.5-8.5), as Cl^-^ depletion reduces transport competition.

**Figure S3.** H_2_ Faradaic efficiency ($\mathrm{FE}_{H_{2}}$) over ten electrocatalytic desalination cycles (2 mL per cycle) at −1.3 V vs. RHE in 1 M NaHCO_3_ electrolyte.


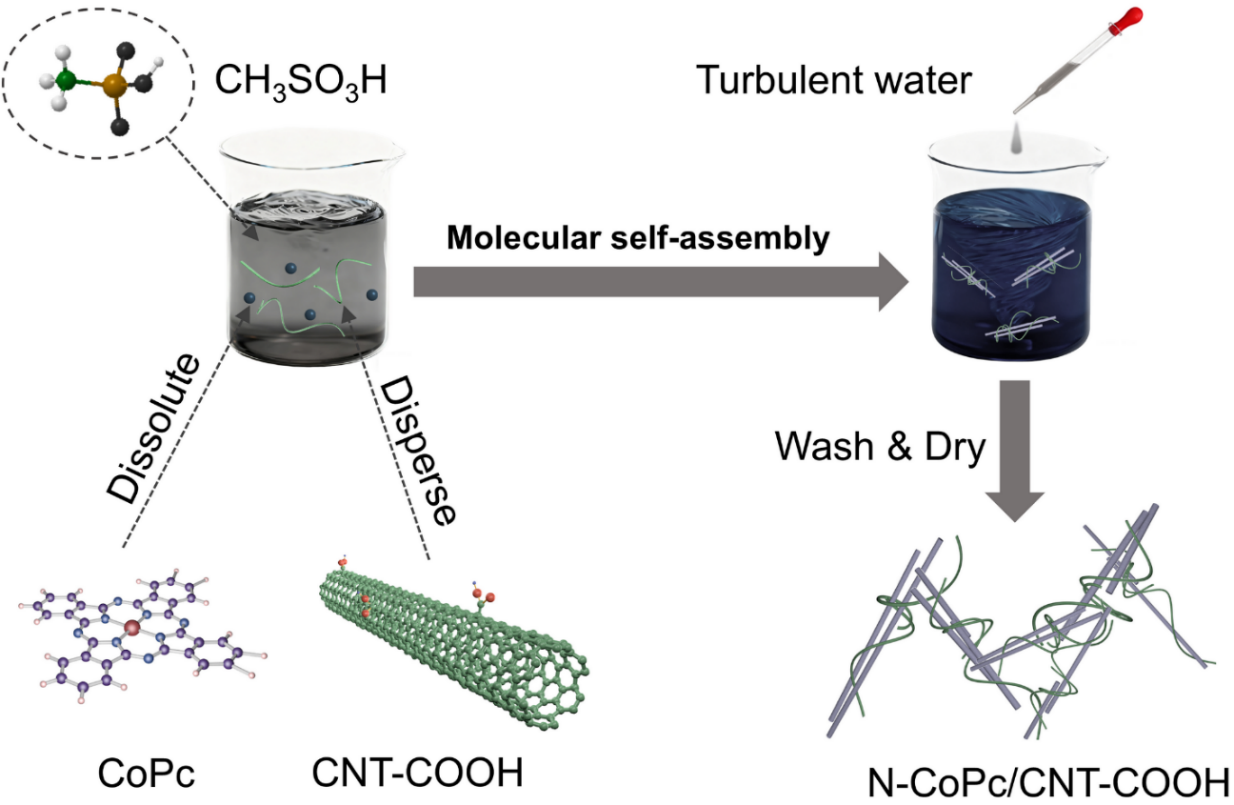


**Figure S4.** Schematic illustration of the synthesis process of N-CoPc/CNT-COOH electrocatalyst.

**Figure S5.** UV-vis-NIR spectra of bulk CoPc, CNT-COOH, and N-CoPc/CNT-COOH.

**Figure S6.** FTIR spectra of bulk CoPc, CNT-COOH, and N-CoPc/CNT-COOH.

**
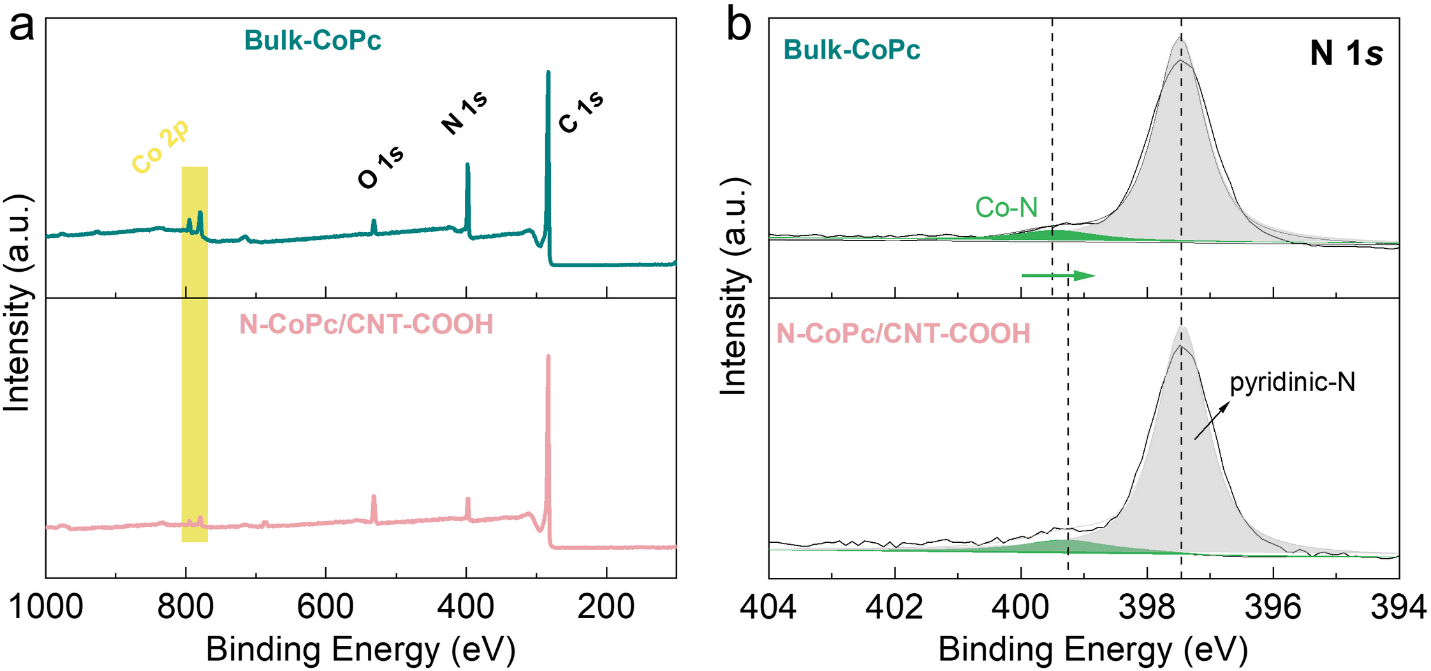
**

**Figure S7.** (a) XPS survey spectra of bulk CoPc and N-CoPc/CNT-COOH; (b) XPS spectra at N 1*s* regions of bulk CoPc and N-CoPc/CNT-COOH**.**

**
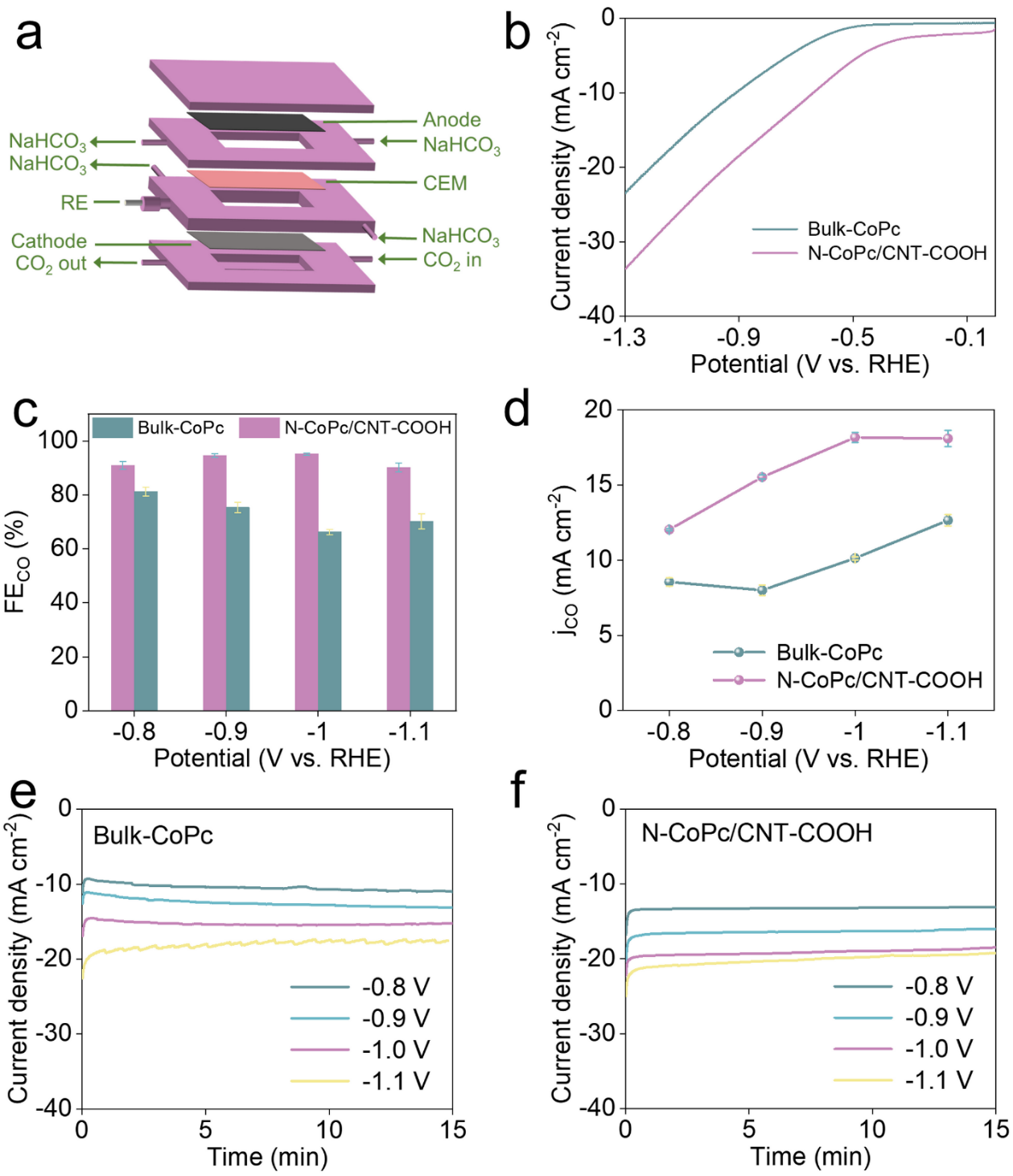
**

**Figure S8.** (a) Schematic illustration of the flow cell used for CO_2_RR in this work; (b) LSV curves; (c) $\mathrm{FE}_{\mathrm{CO}}$; (d) j_CO_ and (e,f) applied potential dependence of total current density of the flow cells using bulk CoPc and N-CoPc/CNT-COOH as catalysts with 1 M NaHCO_3_ electrolyte.

**
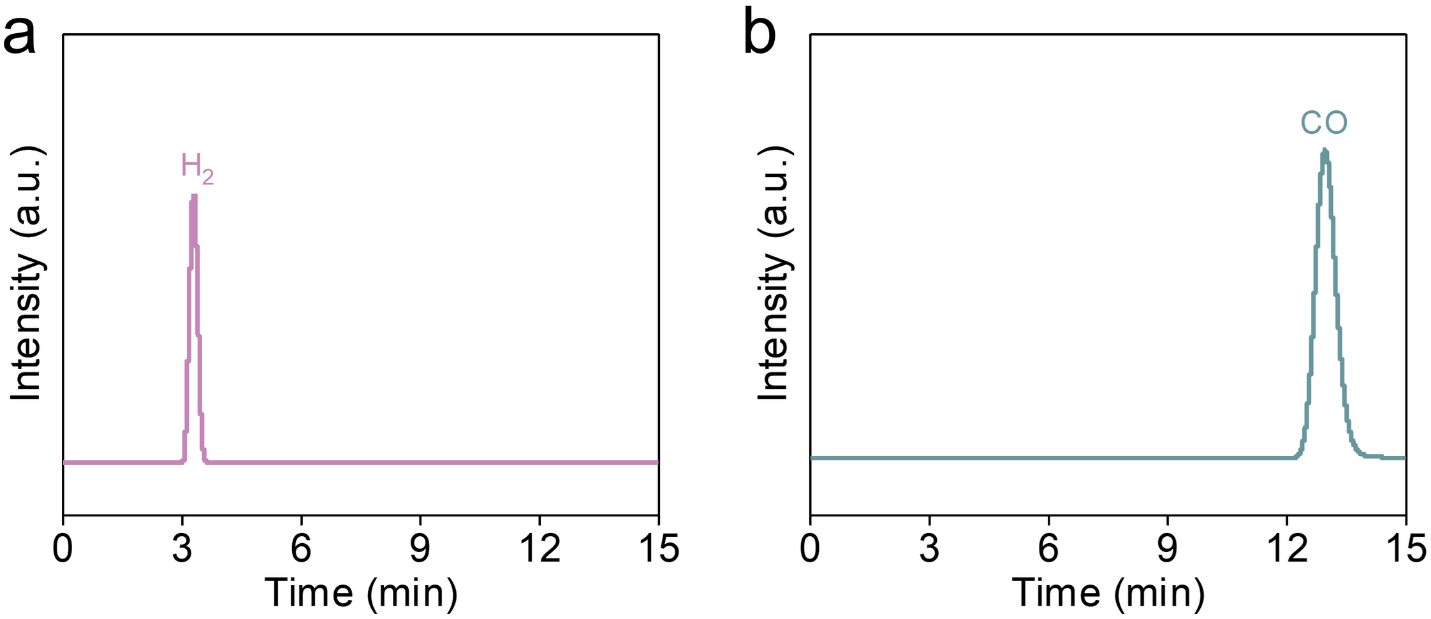
**

**Figure S9.** (a) The H_2_ peak detected by the thermal detector and (b) the CO peak detected by the flame ionization detector in GC.

**Figure S10.** Comparative ^1^H NMR spectra of the electrolyte before and after electrocatalytic desalination.

**
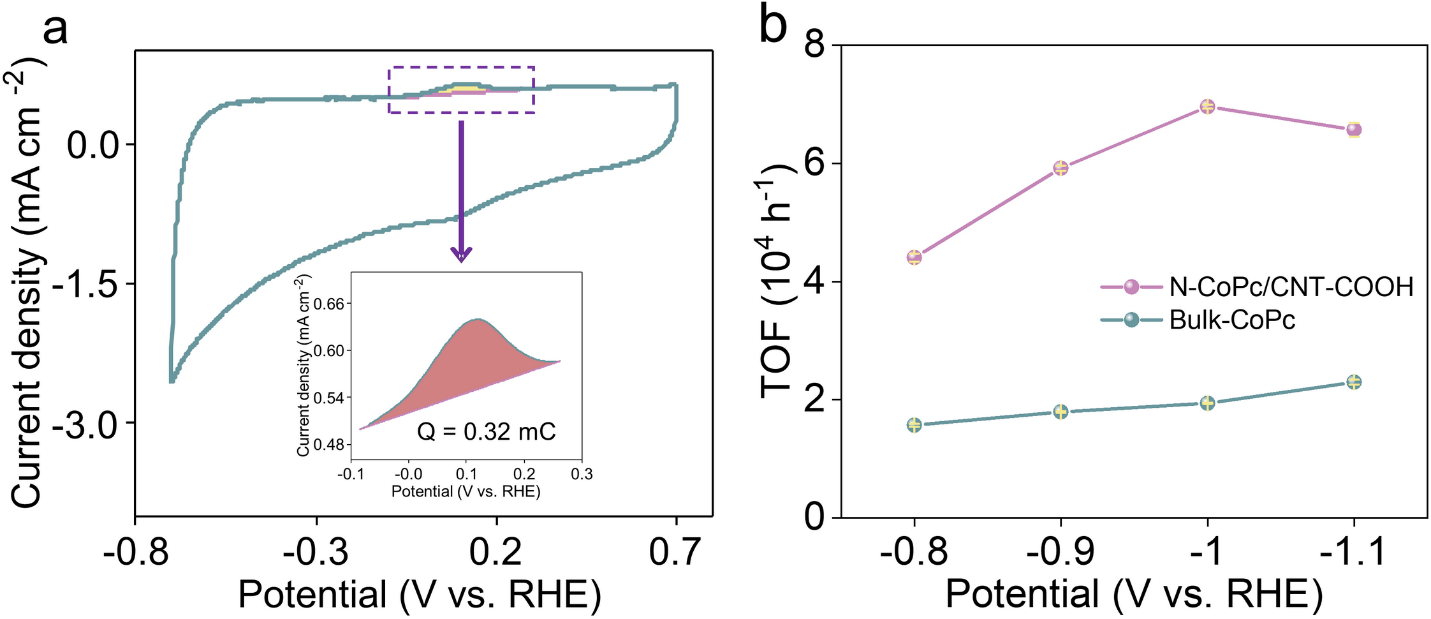
**

**Figure S11.** (a) CV curve of N-CoPc/CNT-COOH in Ar-saturated 1 M NaHCO_3_ aqueous electrolyte (the insert shows the total charge integrated from the Co(II)/Co(I) transition); (b) TOF of N-CoPc/CNT-COOH and bulk CoPc at different potentials.

**
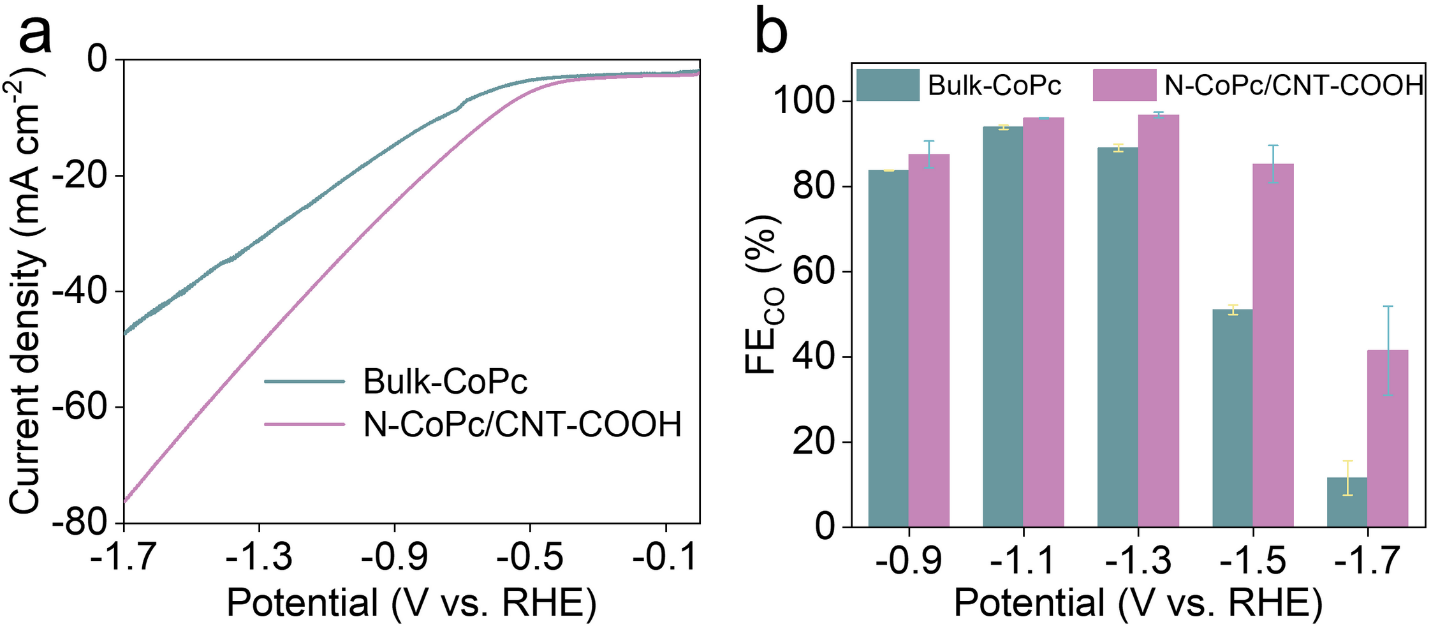
**

**Figure S12.** (a) LSV curves of bulk CoPc and N-CoPc/CNT-COOH and (b) $\mathrm{FE}_{\mathrm{CO}}$ of the electrocatalytic desalination device with 1 M NaHCO_3_ at various potentials.


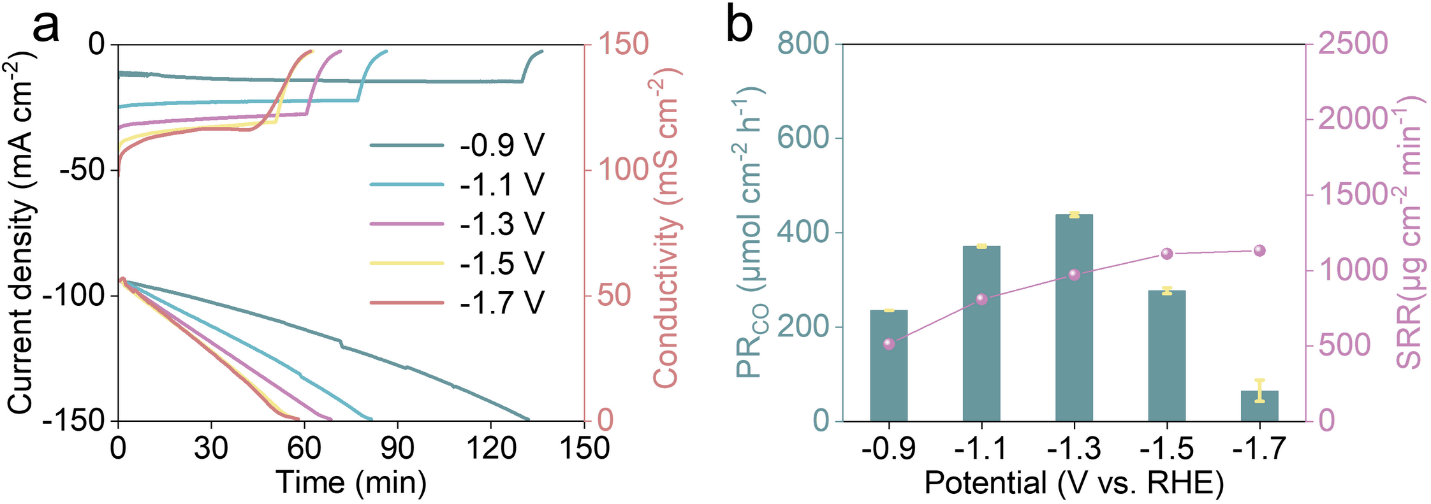


**Figure S13.** (a) The i-t curves and corresponding changes in ionic conductivity; (b) PR_CO_ and SRR for bulk CoPc in the electrocatalytic desalination device at different potentials.

**Figure S14.** Nyquist plots of bulk CoPc and N-CoPc/CNT-COOH in the flow cell with 1 M NaHCO_3_ saturated with CO_2_.


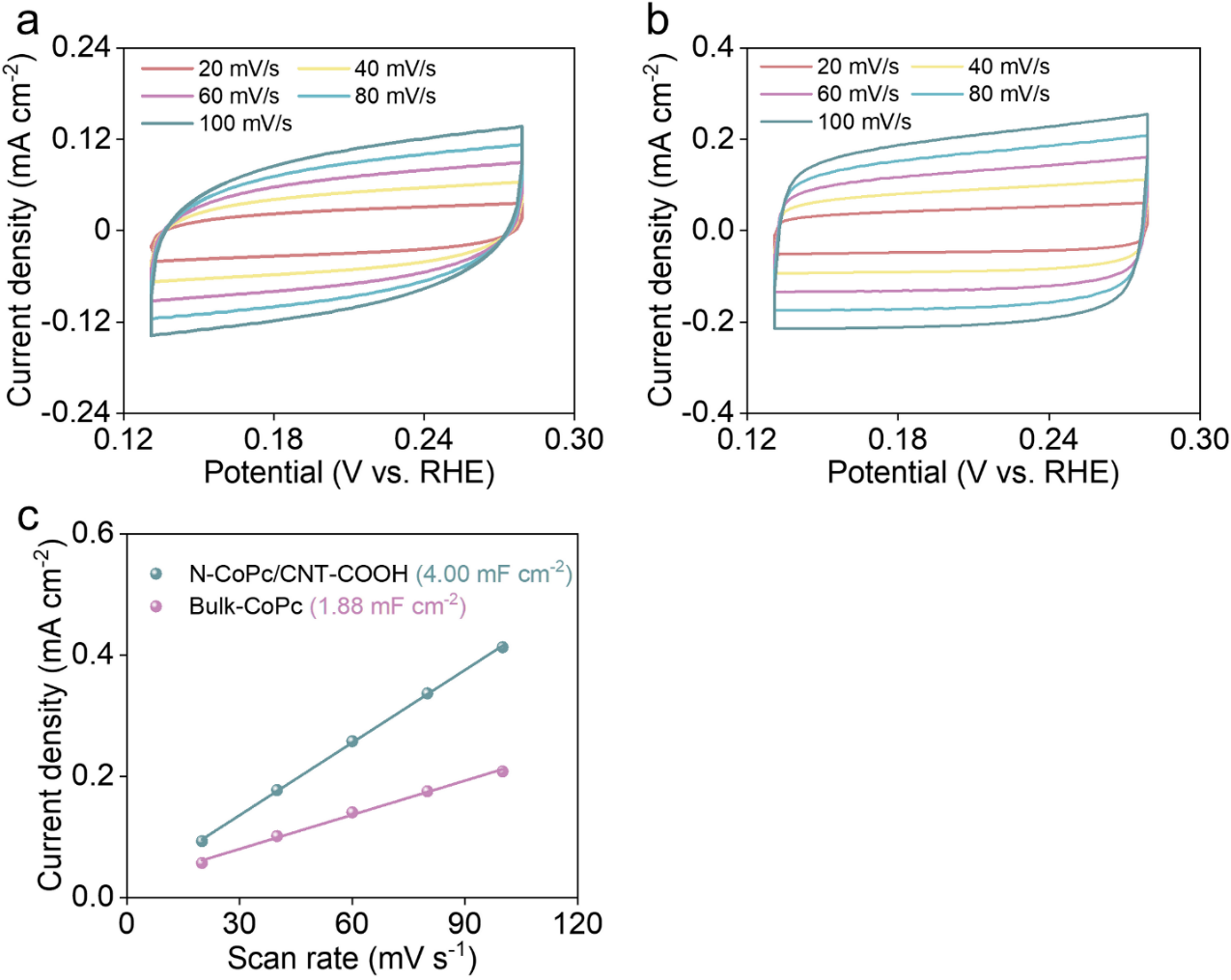


**Figure S15.** CV curves of (a) bulk CoPc; (b) N-CoPc/CNT-COOH collected at different scan rates in 1 M NaHCO_3_; (c) Plots of the current density versus the scan rate for the samples above.

**Figure S16.** Linear correlation between the current density (*j*) and salt removal rate (*SRR*) under different experimental conditions. The data points were collected by varying applied potentials (corresponding to Fig. 3a-c) and electrolyte concentrations (corresponding to Fig. 3g-i). The linear fitting equation and coefficient of determination (R^2^) are indicated.

**Figure S17.** Variation of $\mathrm{FE}_{H_{2}}$ in the electrocatalytic desalination device during a long-term stability test without refreshing its electrolyte (1 M NaHCO_3_).

**Figure S18.** Freshwater production rate (PR_freshwater_) and CO production rate (PR_CO_) of the electrocatalytic desalination device during the long-term stability test.

**Figure S19.** Changes in ionic conductivity and its derivative during the long-term stability test without electrolyte refresh.

**Figure S20.** UV-Vis spectra illustrating the absorbance response from the DPD test, indicating the presence of free chlorine dissolved in the electrolyte after testing.

**
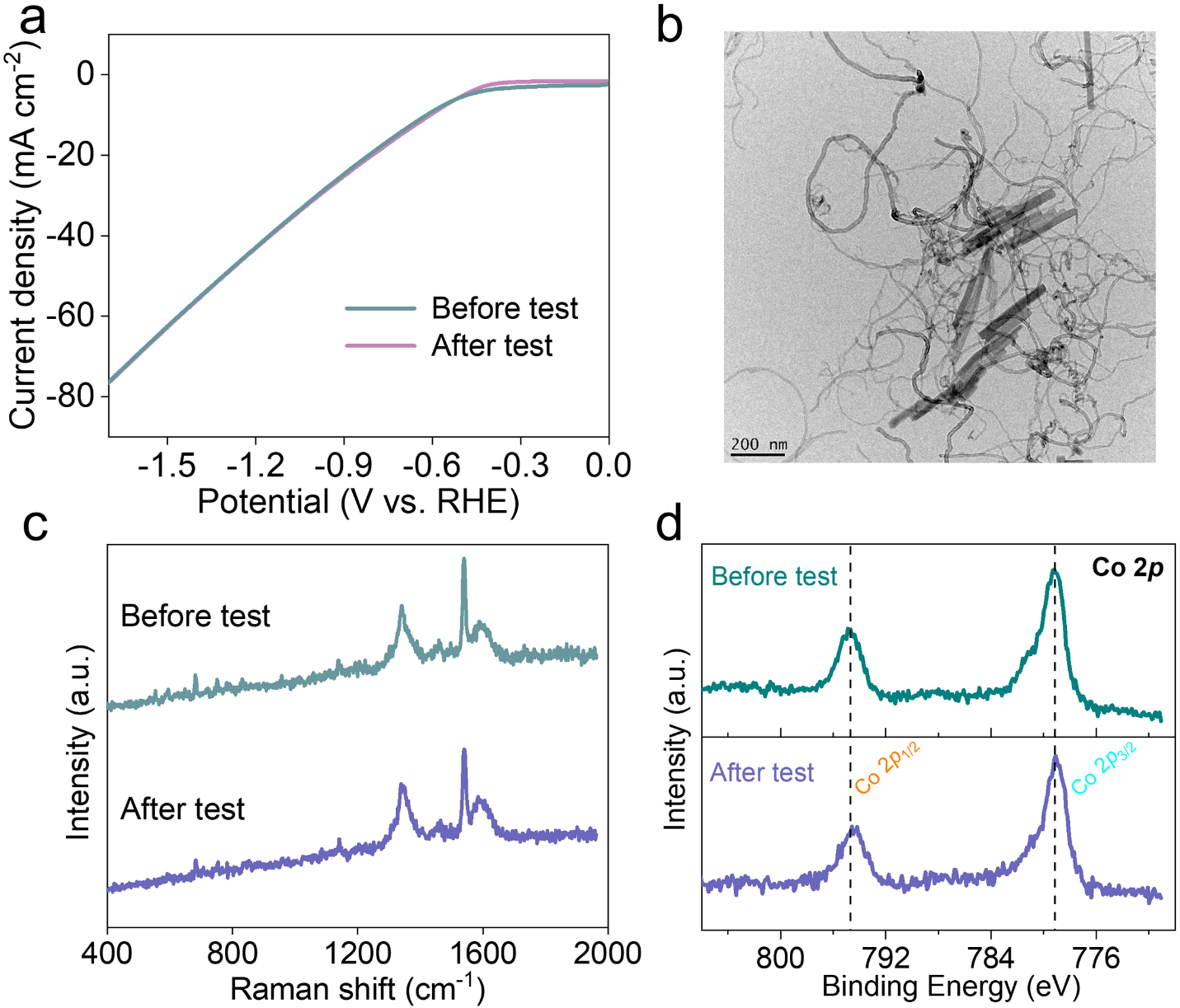
**

**Figure S21.** (a) LSV curves of N-CoPc/CNT-COOH before and after the stability test; (b) TEM image of N-CoPc/CNT-COOH after the stability test; (c) Raman spectra and (d) Co 2*p* XPS spectra of N-CoPc/CNT-COOH before and after the stability test.

**Table S1.** A comparison of the performance of the reported CO_2_-to-CO electrocatalysts in neutral electrolytes.

| **Catalysts** | **Electrolyte** | **E (V vs. RHE)** | ***j*_CO_ (mA cm^-2^)** | ***FE_CO_* (%)** | **Ref.** |
| --- | --- | --- | --- | --- | --- |
| Cu-Sn | 0.1 M KHCO_3_ | -0.75 | 6.2 | 93 | [1] |
| NiPc-NiO_4_ | 0.5 M KHCO_3_ | -0.85 | 8 | 90 | [2] |
| V_0_-CeO_2_/C-1300 | 0.5 M KHCO_3_ | -0.8 | 0.5 | 80 | [3] |
| CoPc-PDQ-COF | 0.5 M KHCO_3_ | -0.66 | 24 | 90 | [4] |
| Zn Dendrite | 0.5 M NaHCO_3_ | -1.1 | 16 | 75 | [5] |
| CCG/CoPc-A Hybrid | 0.1 M KHCO_3_ | -0.69 | 4 | 82 | [6] |
| Nanoporous ZnO | 0.25 M K_2_SO_4_  (PH = 4.2) | -1.14 | 15 | 92 | [7] |
| Ni-SAC (Pc) | 0.5 M KHCO_3_ | -0.64 | 10 | 97 | [8] |
| FNC-SnOF | 0.1 M KHCO_3_ | -0.75 | 8 | 95 | [9] |
| CoPPc/CNT | 0.5 M NaHCO_3_ | -0.56 | 13 | 82 | [10] |
| Zn Nanoflakes | 0.1 M KHCO_3_ | -1.1 | 23 | 48 | [11] |
| CoPc/MAD-OH | 0.1 M KHCO_3_ | -1.0 | 7.5 | 94 | [12] |
| Re-Zn-CO_2_/KCl | 0.5 M KHCO_3_ | -1.05 | 4.3 | 96 | [13] |
| CuPcF_8_-CoNPc-COF | 0.5 M CsHCO_3_ | -0.62 | 15 | 95 | [14] |
| Cu NAs-light | 0.1 M KHCO_3_ | -0.7 | 3.6 | 55 | [15] |
| N-CoMe_2_Pc/NRGO  (6:10) | 0.5 M KHCO_3_ | -0.8 | 15 | 90 | [16] |
| FeMn-N-C | 0.1 M KHCO_3_ | -0.5 | 1.8 | 80 | [17] |
| Fe-N-C |  |  | 3.1 | 79 |  |
| Mn-N-C |  |  | 2.0 | 80 |  |
| Fe-N-C | 0.1 M KHCO_3_ | -0.62 | 6.3 | 78 | [18] |
| Co-u-COF/Graphene | 0.5 M KHCO_3_ | -0.68 | 8 | 92 | [19] |
| COF-367-Co | 0.5 M KHCO_3_ | -0.67 | 2.8 | 89 | [20] |
| ZnAlCe-THs | 0.1 M KHCO_3_ | -1.2 | 10.5 | 89 | [21] |
| CoF_4_Pc/CNT (2:30) | 0.5 M KHCO_3_ | -0.85 | 29 | 92 | [22] |
| Cu-Sb-S-Derived | 1.0 M KHCO_3_ | -1.0 | 38 | 81 | [23] |
| CoPc/GPY/G | 0.1 M KHCO_3_ | -0.81 | 6 | 92 | [24] |
| F-ZL-LDH | 0.1 M KHCO_3_ | -1.3 | 4.6 | 89 | [25] |
| F-ZC-LDH |  |  | 6.0 | 90 |  |
| **N-CoPc/CNT-COOH** | **1.0 M NaHCO_3_** | **-1.0** | **18.3** | **96.6** | **This work** |
|  | **1.0 M NaHCO_3_**  **(Integrated with desalination)** | **-1.3** | **38.2** | **95.5** |  |

**Table S2.** EIS analysis results of the bulk CoPc and N-CoPc/CNT-COOH.

| **Samples** | **R_s_ (Ω)** | **R_ct_ (Ω)** |
| --- | --- | --- |
| bulk CoPc | 5.26 | 8.93 |
| N-CoPc/CNT-COOH | 4.89 | 4.47 |

**Table S3.** Comparison of the initial and final ion contents of real seawater treated by the electrocatalytic device after the first desalination cycle.

| Item | Unit | Initial | Desalted  stream | Removal  efficiency |
| --- | --- | --- | --- | --- |
| Conductivity | mS cm^-1^ | 56.0 | 0.07 | - |
| Na^+^ | mg L^-1^ | 13485.9 | 12.1 | 99.91% |
| K^+^ | mg L^-1^ | 318.5 | 0.12 | 99.96% |
| Mg^2+^ | mg L^-1^ | 17.8 | 0.26 | 98.54% |
| Sr^2+^ | mg L^-1^ | 8.8 | 0.11 | 98.75% |
| Ca^2+^ | mg L^-1^ | 70.9 | 1.40 | 98.03% |
| Cl^-^ | mg L^-1^ | 21095.0 | 19.6 | 99.91% |
| SO_4_^2-^ | mg L^-1^ | 282.3 | 8.03 | 97.16% |

**Table S4.** ICP-OES analysis of cation concentrations and IC analysis of Cl^-^ in the electrolyte before and after the long-term stability test.

| **Item** | **Unit** | **Before test** | **After test** |
| --- | --- | --- | --- |
| Na^+^ | mg L⁻^1^ | 21742 | 21929 |
| K^+^ | mg L⁻^1^ | 0.64 | 6.25 |
| Mg^2+^ | mg L⁻^1^ | 0.30 | 0.84 |
| Sr^2+^ | mg L⁻^1^ | 0.15 | 0.24 |
| Ca^2+^ | mg L⁻^1^ | 3.70 | 4.96 |
| Cl^-^ | mg L⁻^1^ | 0.066 | 0.123 |

**Table S5.** Comparison of different desalination techniques regarding electrode materials, initial salt concentrations, current density, SRR, and SRE.

| **Desalination technology** | **Electrode materials/** | **Applied voltage/**  **Current density** | **Initial salt**  **concentration**  **(ppm)** | **SRR**  **(μg cm^-2^ min^-1^)** | **SRE**  **(%)** | **Reference** |
| --- | --- | --- | --- | --- | --- | --- |
| F-DI | BiOCl \|\| NMO | 0.5 A g^-1^ | 760 | 177 | 1.5 | [26] |
|  | PNDIE | 1.2 V | 3000 | 32.8 | 15.9 | [27] |
|  | Na_0.44_MnO_2_ \|\| Na_0.7_MnO_2+δ_ | 0.8 V | 3000 | 42 | - | [28] |
|  | NiHCF | 1 mA cm^-2^ | ~584 | 29.8 | 90 | [29] |
|  |  |  |  | 31.0 | 80 |  |
|  |  |  | ~1169 | 26.9 | 52 |  |
| RF-DI | [Fe(CN)_6_]^3-/4-^ (100mM) | 0.2 V | 3000 | 65.9 | 95 | [30] |
|  | VCl_3_ (25 mM) NaI (18 mM) | 0.22 mA cm^-2^ | 19000 | 44 | 7.8 | [31] |
|  | TEMPO (4 mM) | 0.44 mA cm^-2^ | ~5860 | 12.1 | 52.4 | [32] |
|  | [BTMAP-Fc]^3+^/^2+^ (50 mM)  [Fe(CN)_6_]^4-^/^3-^ (100 mM) | 0.5 V | 35000 | 633 | 99.5 | [33] |
|  |  |  |  | 880 | 99.2 |  |
|  | NaI/NaI_3_ | 3.0 mA cm^-2^ | 10000 | 63.8 | 89.5 | [34] |
|  | Fe^2+/ 3+^-DPTA (50 mM) | 1.0 V | ~2922 | 39.4 | 85.5 | [35] |
|  | FMN-Na (25mM) | 0.48 mA cm^-2^ | 5200 | 141.6 | 98.1 | [36] |
|  | [Fe(CN)_6_]^3-/4−^ (10 mM)  and I_3_^−^/I^−^ (1 mM) | 5.7 mA cm^-2^ | 4000 | 164.9 | 99.8 | [37] |
|  |  | 7.6 mA cm^-2^ | 32500 | 210 | 99.4 |  |
| Conventional CDI | MWCNTs | 1.2 V | 3000 | 0.45 | 60.7 | [38] |
|  | commercial AC | 1.2 V | ~1169 | 12.8 | 20 | [39] |
|  | OMC | 1.2 V | 25.6 | 7.6 | 57 | [40] |
|  | OMC-S | 0.8 V | 50 | 8.1 | 16.8 | [41] |
|  | AC-QPVP \|\| AC-HNO_3_ | 1.2 V | 500 | 8.2 | 21.4 | [42] |
|  | Ag@hMXene \|\| hMXene | 1.0 V | 1500 | 2.2 | 93 | [43] |
| M-CDI | ACC | 1.2 V | ~584 | 77.7 | 12.8 | [44] |
|  | PANI-CC-AC | 0.8 A | 2000 | 1350 | - | [45] |
| F-CDI | Activated carbon (5wt%)  \|\| FCNTs (0.25w t%) | 1.2 V | 35000 | 1616.5 | 19.0 | [46] |
|  | PANI-Carbon Cloth-MoS_2_  \|\| Ni Foam | 1.8 V | 2000 | 116 | 83 | [47] |
|  | AC/w 300mM HQ | 1.2 V | 500 | 746.9 | 26.3 | [48] |
|  | UCNs@PILs-5 | 1.4V | 3000 | 22.9 | 12.6 | [49] |
|  | Graphite (ICC) | 18 mA cm^-2^ | 1000 | 132 | 85 | [50] |
|  | AC (20wt%) | 4.8 V | 30000 | 208 | ~2.06 | [51] |
|  | AC \|\| ZIF-67@CNTs | 1.2 V | 10000 | 382.6 | 37.3 | [52] |
|  | NVOPF@rGO-AC | 1.6V | 1000 | 5.32 | 32.3 | [53] |
|  | NPC \|\| CB (1wt% carbon content) | 1.2 V | 2000 | 25.8 | 66.9 | [54] |
|  | 3D-Titanium mesh | 2.0 V | 34200 | 36.2 | 97.5 | [55] |
|  | Tip-Array current collector | 1.5 V | 35000 | 3.5 | 99.9 | [56] |
| EC-DI | Bi \|\| Ir/C | 4.6 mA cm^-2^ | 15000 | 228.4 | 99.9 | [57] |
|  | Bi \|\| n-TEC | 6 mA cm^-2^ | 10000 | 722.4 | 97.5 | [58] |
|  |  | 8.5 mA cm^-2^ | 36000 | - | 98.6 |  |
|  | Cu@ZIF-8C \|\| Ir/C | -1.1 V | 35000 | 1043.5 | 99.4 | [59] |
|  | Fe_8_Zn_1_PPc \|\| Ir/C | -1.2 V | 35000 | 1324.4 | 99.1 | [60] |
|  | SnPc/NRGO (1:2) \|\| IrO_2_/Ti | -1.2 V | 35220 | 824 | 99.7 | [61] |
|  | **N-CoPc/CNT-COOH \|\| IrO_2_/Ti** | **-1.3 V**  **~41 mA cm^-2^** | **~35000** | **1592.8** | **99.88** | **This work**  **(continuous)** |

F-DI: faraday deionization; RF-DI: redox flow deionization; CDI: capacitive deionization; M-CDI: membrane capacitive deionization; F-CDI: flow capacitive deionization; EC-DI: electrocatalytic desalination

**Table S6.** Comparison of different desalination techniques: advantages, limitations, suitable feed water, and energy consumption.

| **Technology Type** | **Technology** | **Advantages** | **Limitations** | **Suitable Feed Water** | **Energy Consumption (kWh m⁻^3^)** | **Ref.** |
| --- | --- | --- | --- | --- | --- | --- |
| Membrane-Based | Reverse Osmosis (RO) | 1. High desalination capacity 2. Modularity and scalability 3. Moderate energy use | 1. High fouling potential 2. High-pressure operation 3. Membrane sensitivity and cost | Seawater/ Brackish Water | 0.4-17 | [62–66] |
|  | Forward Osmosis (FO) | 1. Low fouling propensity 2. Low energy driver 3. Avoids the discharge of concentrated brine | 1. Draw the solute regeneration challenge 2. Low desalination rate 3. Limited membrane availability | Seawater/ Brackish Water | 0.01-0.4 | [67–70] |
| Thermal | Multistage Flash Distillation (MSF) | 1. Reliability and large capacity 2. Tolerance to feed water quality 3. High-temperature operation | 1. High energy consumption 2. High capital and operating costs 3. Slow startup and response time | Seawater | 20-87 | [71–74] |
|  | Multi-effect Distillation (MED) | 1. Higher thermal efficiency 2. Greater operational flexibility 3. High product purity | 1. Scaling and corrosion 2. Complex maintenance 3. High capital cost | Seawater | 14-21 | [75–79] |
|  | Membrane Distillation (MD) | 1. Low-temperature operation 2. Theoretical high rejection | 1. Low water flux and efficiency 2. Membrane wetting and fouling | Seawater | 49-801 | [80–83] |
|  | Freezing Desalination  (FD) | 1. Effective for hypersaline water 2. Corrosion-free operation | 1. Complex ice-crystal separation 2. High refrigeration costs | Seawater/  Hypersaline | 7-173 | [84–86] |
|  | Solar Distillation (SD) | 1. Zero operational emissions 2. Minimal maintenance | 1. Evaporator salt tolerance concern 2. Large land footprint 3. Weather-dependent output | Seawater/ Brackish Water | Weather-dependent | [87–89] |
| Microbial | Microbial Desalination Cell (MDC) | 1. Energy-positive process 2. Low energy consumption 3. Environmentally friendly operation | 1. Low and unstable desalination rate 2. System complexity and pH imbalance 3. Limited salinity reduction and scalability | Salt-containing Wastewater | Archebiosis | [90–93] |
| Electrochemical | Electrodialysis (ED) | 1. Scalable modular design 2. Energy efficient for brackish water | 1. Membrane degradation and cost 2. Pretreatment needs 3. High energy consumption for seawater | Seawater/  Brackish Water | 1.2-15 | [94–98] |
|  | Faradaic Deionization  (F-DI) | 1. Avoidance of Co-ion Expulsion 2. high salinity tolerance 3. Lower energy consumption potential | 1. Poor electrode cyclability and stability 2. Complex and costly material synthesis 3. Potential for soluble side product | Brackish Water | 0.02-0.55 | [99–103] |
|  | Redox Flow Deionization  (RF-DI) | 1. Continuous and stable operation 2. Eco-friendly operation 3. Decoupled energy and power | 1. System complexity 2. Electrolyte crossover and degradation 3. Lower energy efficiency at high current density | Brackish Water | 0.1-5.7 | [104–108] |
|  | Capacitive Deionization (CDI) | 1. Low energy consumption 2. Environmental friendliness 3. Simple electrode regeneration | 1. Electrode degradation 2. Unsuitable for high salinity 3. Co-ion expulsion and limited capacity | Brackish Water | 0.1-2 | [109–112] |
|  | Flow Electrode CDI (F-CDI) | 1. Continuous operation without regeneration 2. Unlimited salt removal capacity 3. High salinity tolerance | 1. Electrode sedimentation and clogging 2. Kinetic limitations and ohmic losses | Seawater/ Brackish Water | 0.1-14 | [113–117] |
|  | Membrane CDI  (M-CDI) | 1. Deeper desalination 2. Mitigated electrode fouling | 1. Increased cost and complexity 2. Discontinuous operation | Brackish Water | 0.1-1.1 | [118–122] |
|  | **Electrocatalytic Desalination (EC-DI)** | 1. **Industrial potential based on a high desalination rate** 2. **Value-added co-products** 3. **Continuous cyclic operation** 4. **Suitable for different catalytic reactions and process scenarios** | 1. **Additional energy consumption is required to produce additional products** 2. **Pretreatment Requirements** 3. **Rely on catalyst performance** | **Seawater/**  **Brackish Water** | **~8.57** | **This work**  **(continuous)** |

**Table S7**. Daily operational cost analysis for electrolyte and membrane replacement (1 m^2^ cross-sectional area). Costs are calculated under identical operating conditions to highlight the economic impact of the electrolyte and membrane used in this work relative to literature benchmarks.

| Electrolytes | pH | Solute consumption  (kg/ day) | No. of Membranes | Membrane service life (year) | Electrolyte cost ($/day) | Membrane Cost ($/day) | Total cost ($/day) | Ref. |
| --- | --- | --- | --- | --- | --- | --- | --- | --- |
| 1 M NaHCO_3_ | 8.3 | 0.00079 | 3 | 2 | 0.013 | 0.169 | 0.182 | This work |
| 0.5 M NaHCO_3_ | 8.4 | 4.79 | 2 | 2 | 8.14 | 0.11 | 8.25 | [59] |
| 0.5 M Na_2_SO_4_ + 0.1 M NaNO_3_ | 7.0 | 3.85 + 2.23 | 2 | 2 | 6.08 | 0.11 | 6.19 | [60] |
| 1 M KOH | 14.0 | 5.43 | 2 | 0.5 | 34.75 | 0.45 | 35.20 | [61] |

Membranes (AEM/CEM): 41.1 $/m² (Hangzhou Iontech Environmental Technology Co., Ltd.)

NaHCO_3_ (≥99%): 1,700 $/ton (Shandong Jiuchong Chemical Co., Ltd.)

KOH (≥99%): 6,400 $/ton (Shandong Jianen Chemical Co., Ltd.)

Na_2_SO_4_ (≥99%): 347 $/ton (Weifang Changs Chemical Industry Co., Ltd.)

NaNO_3_ (≥99%): 1,680 $/ton (Shandong Haihua Hualong New Materials Co., Ltd.)

**Table S8.** Breakdown of material costs and amortized daily costs for the device components (1 m^2^ cross-sectional area).

| Materials | Price | Source | | Quantity | | Daily cost for 15-year service life | |
| --- | --- | --- | --- | --- | --- | --- | --- |
| Acrylic plates | 7.40 $/m^2^ | | Anhui Xintao Photoelectric Technology Co., Ltd. | | 6 m^2^ | | 0.0081 $ |
| Bolts | 0.52 $/unit | | Jiaxing Goshen Hardware Co., Ltd. | | 10 units | | 0.0009 $ |
| IrO_2_/Ti | 1287.55 $/m^2^ | | Suzhou High-Tech District Yileka Metal Materials Trading Dept. | | 1 m^2^ | | 0.2352 $ |
| Silica gel plate | 28.61 $/m^2^ | | Anhui Bide New Materials Co., Ltd. | | 5 m^2^ | | 0.0261 $ |
| Peristaltic pump | 599.78 $/unit | | Baoding Signal Fluid Technology Co., Ltd. | | 1 unit | | 0.1095 $ |
| Fluorine rubber hose | 1.53 $/m | | Qray Material Technology Co., Ltd. | | 20 m | | 0.0055 $ |
| Total |  |  | |  | | 0.3855 $ | |

**Table S9.** Detailed cost breakdown of cathode materials and amortized daily replacement costs (based on a 2-year service life).

| Materials | Purity | Source | Price | Amount for each replacement | Daily cost for 2-year service life |
| --- | --- | --- | --- | --- | --- |
| Cobalt(II) phthalocyanine | ≥95% | Shanghai Aladdin Biochemical Technology Co., Ltd. | 5.15 $/g | 2.67 g | 0.0188 $ |
| Methanesulfonic acid | ≥98% | Shanghai Aladdin Biochemical Technology Co., Ltd. | 0.03 $/g | 1.97 g | 0.0008 $ |
| CNT-COOH | XFM03 | Nanjing XFNANO Materials Tech | 3.39 $/g | 5.33 g | 0.0248 $ |
| Carbon paper | Sigracet 36BB | SCI Materials Hub | 214.59 $/m^2^ | 1 m^2^ | 0.2940 $ |
| 5% Nafion solution | D521CS | Shanghai Aladdin Biochemical Technology Co., Ltd. | 3.04 $/ml | 200 ml | 0.8332 $ |
| Total |  |  |  |  | 1.1708 $ |

**Table S10.** Regional techno-economic analysis of CO production costs, including electricity/water tariffs and potential profits from seawater desalination.

| Region | p_ec_ ($/kWh) | p_wa_ ($/ton) | PC_electricity_ ($/ton_CO_) | PC_total_ ($/ton_CO_) | PF_desalin_ ($/ton_CO_) | NC_CO_ ($/ton_CO_) |
| --- | --- | --- | --- | --- | --- | --- |
| Guangdong | 0.072 | 0.63 | 749 | 1191 | 81 | 1111 |
| Inner Mongolia | 0.040 | 1.00 | 416 | 858 | 129 | 730 |
| St.Petersburg | 0.077 | 0.50 | 801 | 1244 | 64 | 1180 |
| Myanmar | 0.023 | 0.32 | 239 | 682 | 41 | 641 |
| Oman | 0.036 | 3.43 | 375 | 817 | 441 | 374 |
| Egypt | 0.021 | 0.17 | 219 | 661 | 22 | 639 |
| Saudi Arabia | 0.053 | 1.37 | 551 | 994 | 176 | 818 |
| Florida | 0.10 | 2.40 | 1040 | 1483 | 309 | 1175 |
| California | 0.15 | 3.65 | 1560 | 2003 | 469 | 1534 |
| Germany | 0.29 | 2.54 | 2997 | 3440 | 327 | 3113 |
| Benchmark | 0.03^[128]^ | 6.36^[129]^ | 312 | 756 | 818 | –63 |

The CO production cost is 600 $/ton_CO_.^[126–128]^

Regional freshwater prices are sourced from the Global Water Intelligence (GWI) report 'The Global Water Tariff Survey 2025', supplemented by data from the International Water Association (IWA) official website and regional government announcements. Regional electricity prices are collected from the International Energy Agency (IEA) report 'Electricity 2026', GlobalPetrolPrices.com, and regional government announcements. Notably, the penultimate row in the table serves as a methodological benchmark for comparative analysis. Specifically, the cost coefficient of 0.03 $/kWh for the CO_2_RR process constitutes a universally accepted assumption in economic assessments of CO_2_ reduction technologies,^[128]^ reflecting a standardized electricity price proxy that transcends regional variations. Similarly, the seawater desalination cost of 6.36 $/ton_freshwater_ is anchored to the well-established reference cost of Reverse Osmosis (RO) desalination,^[129]^ rather than site-specific fresh water prices or regional cost structures. This deliberate choice aims to decouple the technological performance of the electrocatalytic system from external economic factors, enabling direct comparison across heterogeneous geographical contexts and highlighting the intrinsic cost-efficiency potential of the integrated CO_2_RR-desalination system.

**References cited in the Supporting Information:**

[1] J. Zeng, K. Bejtka, W. Ju, M. Castellino, A. Chiodoni, A. Sacco, M.A. Farkhondehfal, S. Hernández, D. Rentsch, C. Battaglia, C.F. Pirri, Advanced Cu-Sn foam for selectively converting CO2 to CO in aqueous solution, Appl. Catal. B Environ. 236 (2018) 475–482. https://doi.org/10.1016/j.apcatb.2018.05.056.

[2] J. Yi, D. Si, R. Xie, Q. Yin, M. Zhang, Q. Wu, G. Chai, Y. Huang, R. Cao, Conductive Two‐Dimensional Phthalocyanine‐based Metal–Organic Framework Nanosheets for Efficient Electroreduction of CO _2_, Angew. Chem. Int. Ed. 60 (2021) 17108–17114. https://doi.org/10.1002/anie.202104564.

[3] Y. Wu, Y. Chang, J. Jia, Studying CeO_2_ -modified defective carbon as an electrocatalyst for electrochemical reduction of CO_2_, RSC Adv. 15 (2025) 4562–4572. https://doi.org/10.1039/D4RA08845J.

[4] N. Huang, K.H. Lee, Y. Yue, X. Xu, S. Irle, Q. Jiang, D. Jiang, A Stable and Conductive Metallophthalocyanine Framework for Electrocatalytic Carbon Dioxide Reduction in Water, Angew. Chem. Int. Ed. 59 (2020) 16587–16593. https://doi.org/10.1002/anie.202005274.

[5] J. Rosen, G.S. Hutchings, Q. Lu, R.V. Forest, A. Moore, F. Jiao, Electrodeposited Zn Dendrites with Enhanced CO Selectivity for Electrocatalytic CO_2_ Reduction, ACS Catal. 5 (2015) 4586–4591. https://doi.org/10.1021/acscatal.5b00922.

[6] J. Choi, P. Wagner, S. Gambhir, R. Jalili, D.R. MacFarlane, G.G. Wallace, D.L. Officer, Steric Modification of a Cobalt Phthalocyanine/Graphene Catalyst To Give Enhanced and Stable Electrochemical CO _2_ Reduction to CO, ACS Energy Lett. 4 (2019) 666–672. https://doi.org/10.1021/acsenergylett.8b02355.

[7] X. Jiang, F. Cai, D. Gao, J. Dong, S. Miao, G. Wang, X. Bao, Electrocatalytic reduction of carbon dioxide over reduced nanoporous zinc oxide, Electrochem. Commun. 68 (2016) 67–70. https://doi.org/10.1016/j.elecom.2016.05.003.

[8] Y. Wang, Z. Jiang, X. Zhang, Z. Niu, Q. Zhou, X. Wang, H. Li, Z. Lin, H. Zheng, Y. Liang, Metal Phthalocyanine-Derived Single-Atom Catalysts for Selective CO_2_ Electroreduction under High Current Densities, ACS Appl. Mater. Interfaces 12 (2020) 33795–33802. https://doi.org/10.1021/acsami.0c08940.

[9] W. Ni, Y. Gao, Y. Lin, C. Ma, X. Guo, S. Wang, S. Zhang, Nonnitrogen Coordination Environment Steering Electrochemical CO _2_ -to-CO Conversion over Single-Atom Tin Catalysts in a Wide Potential Window, ACS Catal. 11 (2021) 5212–5221. https://doi.org/10.1021/acscatal.0c05514.

[10] N. Han, Y. Wang, L. Ma, J. Wen, J. Li, H. Zheng, K. Nie, X. Wang, F. Zhao, Y. Li, J. Fan, J. Zhong, T. Wu, D.J. Miller, J. Lu, S.-T. Lee, Y. Li, Supported Cobalt Polyphthalocyanine for High-Performance Electrocatalytic CO2 Reduction, Chem 3 (2017) 652–664. https://doi.org/10.1016/j.chempr.2017.08.002.

[11] B. Qin, Y. Li, H. Fu, H. Wang, S. Chen, Z. Liu, F. Peng, Electrochemical Reduction of CO_2_ into Tunable Syngas Production by Regulating the Crystal Facets of Earth-Abundant Zn Catalyst, ACS Appl. Mater. Interfaces 10 (2018) 20530–20539. https://doi.org/10.1021/acsami.8b04809.

[12] F. Yu, Z. Zhou, Y. You, J. Zhan, T. Yao, L.-H. Zhang, Tuning the Hydroxyl Density of MXene to Regulate the Electrochemical Performance of Anchored Cobalt Phthalocyanine for CO _2_ Reduction, ACS Appl. Mater. Interfaces 15 (2023) 24346–24353. https://doi.org/10.1021/acsami.3c01012.

[13] D.L.T. Nguyen, M.S. Jee, D.H. Won, H. Jung, H.-S. Oh, B.K. Min, Y.J. Hwang, Selective CO_2_ Reduction on Zinc Electrocatalyst: The Effect of Zinc Oxidation State Induced by Pretreatment Environment, ACS Sustain. Chem. Eng. 5 (2017) 11377–11386. https://doi.org/10.1021/acssuschemeng.7b02460.

[14] Y. Yue, P. Cai, K. Xu, H. Li, H. Chen, H.-C. Zhou, N. Huang, Stable Bimetallic Polyphthalocyanine Covalent Organic Frameworks as Superior Electrocatalysts, J. Am. Chem. Soc. 143 (2021) 18052–18060. https://doi.org/10.1021/jacs.1c06238.

[15] J. Xue, Z. Chen, K. Dang, L. Wu, H. Ji, C. Chen, Y. Zhang, J. Zhao, The plasmonic effect of Cu on tuning CO_2_ reduction activity and selectivity, Phys. Chem. Chem. Phys. 26 (2024) 2915–2925. https://doi.org/10.1039/D3CP05450K.

[16] M. Li, C. Yan, R. Ramachandran, Y. Lan, H. Dai, H. Shan, X. Meng, D. Cui, F. Wang, Z.-X. Xu, Non-peripheral octamethyl-substituted cobalt phthalocyanine nanorods supported on N-doped reduced graphene oxide achieve efficient electrocatalytic CO2 reduction to CO, Chem. Eng. J. 430 (2022) 133050. https://doi.org/10.1016/j.cej.2021.133050.

[17] A.S. Varela, N. Ranjbar Sahraie, J. Steinberg, W. Ju, H. Oh, P. Strasser, Metal‐Doped Nitrogenated Carbon as an Efficient Catalyst for Direct CO_2_ Electroreduction to CO and Hydrocarbons, Angew. Chem. Int. Ed. 54 (2015) 10758–10762. https://doi.org/10.1002/anie.201502099.

[18] A.S. Varela, M. Kroschel, N.D. Leonard, W. Ju, J. Steinberg, A. Bagger, J. Rossmeisl, P. Strasser, pH Effects on the Selectivity of the Electrocatalytic CO_2_ Reduction on Graphene-Embedded Fe–N–C Motifs: Bridging Concepts between Molecular Homogeneous and Solid-State Heterogeneous Catalysis, ACS Energy Lett. 3 (2018) 812–817. https://doi.org/10.1021/acsenergylett.8b00273.

[19] H. Gu, G. Shi, L. Zhong, L. Liu, H. Zhang, C. Yang, K. Yu, C. Zhu, J. Li, S. Zhang, C. Chen, Y. Han, S. Li, L. Zhang, A Two-Dimensional van der Waals Heterostructure with Isolated Electron-Deficient Cobalt Sites toward High-Efficiency CO _2_ Electroreduction, J. Am. Chem. Soc. 144 (2022) 21502–21511. https://doi.org/10.1021/jacs.2c07601.

[20] S. Lin, C.S. Diercks, Y.-B. Zhang, N. Kornienko, E.M. Nichols, Y. Zhao, A.R. Paris, D. Kim, P. Yang, O.M. Yaghi, C.J. Chang, Covalent organic frameworks comprising cobalt porphyrins for catalytic CO_2_ reduction in water, Science 349 (2015) 1208–1213. https://doi.org/10.1126/science.aac8343.

[21] F. Tan, T. Liu, E. Liu, Y. Zhang, On ZnAlCe-THs Nanocomposites Electrocatalysts for Electrocatalytic Carbon Dioxide Reduction to Carbon Monoxide, Catal. Lett. 154 (2024) 11–22. https://doi.org/10.1007/s10562-023-04302-5.

[22] M. Li, J. Xu, F. Qi, Y. Wang, C. Yan, J. Xu, Facile preparation of tetrafluoro-substituted cobalt phthalocyanine nanorods attached on carbon nanotubes for efficient electrocatalytic CO2 reduction, J. Solid State Electrochem. 27 (2023) 1269–1278. https://doi.org/10.1007/s10008-023-05480-3.

[23] D.Y.Y. Goh, K.M. Yam, L. Rekhi, A.D. Handoko, Y.C. Tan, Y. Wang, J.M.R. Tan, T.S. Choksi, Y. Lum, L.H. Wong, Covalency-aided electrochemical CO_2_ reduction to CO on sulfide-derived Cu–Sb, J. Mater. Chem. A 12 (2024) 1840–1851. https://doi.org/10.1039/D3TA04777F.

[24] H. Gu, L. Zhong, G. Shi, J. Li, K. Yu, J. Li, S. Zhang, C. Zhu, S. Chen, C. Yang, Y. Kong, C. Chen, S. Li, J. Zhang, L. Zhang, Graphdiyne/Graphene Heterostructure: A Universal 2D Scaffold Anchoring Monodispersed Transition-Metal Phthalocyanines for Selective and Durable CO _2_ Electroreduction, J. Am. Chem. Soc. 143 (2021) 8679–8688. https://doi.org/10.1021/jacs.1c02326.

[25] J. Wang, T. Liu, R. Xu, Y. Zhang, Effect of F Doping on CO2 Electrocatalytic Performance of Zinc-Based Rare Earth Layered Double Hydroxides, Catal. Lett. 154 (2024) 2687–2700. https://doi.org/10.1007/s10562-023-04526-5.

[26] F. Chen, Y. Huang, L. Guo, L. Sun, Y. Wang, H.Y. Yang, Dual-ions electrochemical deionization: a desalination generator, Energy Environ. Sci. 10 (2017) 2081–2089. https://doi.org/10.1039/C7EE00855D.

[27] A. Fombona-Pascual, N. Patil, E. García-Quismondo, N. Goujon, D. Mecerreyes, R. Marcilla, J. Palma, J.J. Lado, A high performance all-polymer symmetric faradaic deionization cell, Chem. Eng. J. 461 (2023) 142001. https://doi.org/10.1016/j.cej.2023.142001.

[28] A. Fombona-Pascual, S. Pinilla, I. Hormigos, J. Palma, J.J. Lado, Sodium-Manganese Oxides in Faradaic Desalination: Achieving Long-Cycling Stability Through Morphological and Structural Optimization, ENERGY Environ. Mater. 8 (2025) e70022. https://doi.org/10.1002/eem2.70022.

[29] E.R. Reale, L. Regenwetter, A. Agrawal, B. Dardón, N. Dicola, S. Sanagala, K.C. Smith, Low porosity, high areal-capacity Prussian blue analogue electrodes enhance salt removal and thermodynamic efficiency in symmetric Faradaic deionization with automated fluid control, Water Res. X 13 (2021) 100116. https://doi.org/10.1016/j.wroa.2021.100116.

[30] C.-Y. Cheng, T.-H. Chen, K.-Y. Chen, J. Ma, C.-H. Hou, Redox-flow battery with four-channel architecture for continuous and efficient desalination over a wide salinity working range, Desalination 534 (2022) 115783. https://doi.org/10.1016/j.desal.2022.115783.

[31] X. Hou, Q. Liang, X. Hu, Y. Zhou, Q. Ru, F. Chen, S. Hu, Coupling desalination and energy storage with redox flow electrodes, Nanoscale 10 (2018) 12308–12314. https://doi.org/10.1039/C8NR02737D.

[32] J. Wang, Q. Zhang, F. Chen, X. Hou, Z. Tang, Y. Shi, P. Liang, D.Y.W. Yu, Q. He, L.-J. Li, Continuous desalination with a metal-free redox-mediator, J. Mater. Chem. A 7 (2019) 13941–13947. https://doi.org/10.1039/C9TA02594D.

[33] E.S. Beh, M.A. Benedict, D. Desai, J.B. Rivest, A Redox-Shuttled Electrochemical Method for Energy-Efficient Separation of Salt from Water, ACS Sustain. Chem. Eng. 7 (2019) 13411–13417. https://doi.org/10.1021/acssuschemeng.9b02720.

[34] P. Lin, T. Yang, Z. Li, W. Xia, X. Xuan, X. Sun, S.M. Alshehri, T. Ahamad, Y. Yamauchi, X. Xu, Y. Bando, Ion transport channels in redox flow deionization enable ultra-high desalination performance, Nano Energy 102 (2022) 107652. https://doi.org/10.1016/j.nanoen.2022.107652.

[35] R. Xie, D. Yue, Z. Peng, X. Wei, Achieving Energy-Saving, Continuous Redox Flow Desalination with Iron Chelate Redoxmers, Energy Mater. Adv. 4 (2023) 0009. https://doi.org/10.34133/energymatadv.0009.

[36] Q. Zhang, S.H. Aung, T.Z. Oo, F. Chen, Continuous electrochemical deionization by utilizing the catalytic redox effect of environmentally friendly riboflavin-5’-phosphate sodium, Mater. Today Commun. 23 (2020) 100921. https://doi.org/10.1016/j.mtcomm.2020.100921.

[37] X. Wang, M. Liang, J. Zhang, X. Chen, M. Zaw, T.Z. Oo, N.W. Lwin, S.H. Aung, Y. Chen, F. Chen, Double-photoelectrode redox desalination of seawater, Water Res. 239 (2023) 120051. https://doi.org/10.1016/j.watres.2023.120051.

[38] K. Dai, L. Shi, J. Fang, D. Zhang, B. Yu, NaCl adsorption in multi-walled carbon nanotubes, Mater. Lett. 59 (2005) 1989–1992. https://doi.org/10.1016/j.matlet.2005.01.042.

[39] R. Zhao, P.M. Biesheuvel, H. Miedema, H. Bruning, A. Van Der Wal, Charge Efficiency: A Functional Tool to Probe the Double-Layer Structure Inside of Porous Electrodes and Application in the Modeling of Capacitive Deionization, J. Phys. Chem. Lett. 1 (2010) 205–210. https://doi.org/10.1021/jz900154h.

[40] L. Zou, L. Li, H. Song, G. Morris, Using mesoporous carbon electrodes for brackish water desalination, Water Res. 42 (2008) 2340–2348. https://doi.org/10.1016/j.watres.2007.12.022.

[41] L. Li, L. Zou, H. Song, G. Morris, Ordered mesoporous carbons synthesized by a modified sol–gel process for electrosorptive removal of sodium chloride, Carbon 47 (2009) 775–781. https://doi.org/10.1016/j.carbon.2008.11.012.

[42] T. Wu, G. Wang, F. Zhan, Q. Dong, Q. Ren, J. Wang, J. Qiu, Surface-treated carbon electrodes with modified potential of zero charge for capacitive deionization, Water Res. 93 (2016) 30–37. https://doi.org/10.1016/j.watres.2016.02.004.

[43] W. Kong, X. Lu, K. Tan, Y. Wang, B. Nie, M. Zhang, S. Zhao, X. Zhang, Y. Feng, Controlling electrode-potential distribution to enable oxidation stability of MXene-based CDI desalination cells, Water Res. 284 (2025) 123948. https://doi.org/10.1016/j.watres.2025.123948.

[44] N. Kim, S.P. Hong, J. Lee, C. Kim, J. Yoon, High-Desalination Performance via Redox Couple Reaction in the Multichannel Capacitive Deionization System, ACS Sustain. Chem. Eng. 7 (2019) 16182–16189. https://doi.org/10.1021/acssuschemeng.9b03121.

[45] R. Chen, X. Liu, M. Wang, Y. Shu, M. Zhang, B. Liu, Z. Wang, A novel two-stage continuous capacitive deionization system with connected flow electrode and freestanding electrode, Chem. Eng. J. 491 (2024) 152133. https://doi.org/10.1016/j.cej.2024.152133.

[46] Y. Cho, C.-Y. Yoo, S.W. Lee, H. Yoon, K.S. Lee, S. Yang, D.K. Kim, Flow-electrode capacitive deionization with highly enhanced salt removal performance utilizing high-aspect ratio functionalized carbon nanotubes, Water Res. 151 (2019) 252–259. https://doi.org/10.1016/j.watres.2018.11.080.

[47] R. Chen, X. Liu, M. Wang, Y. Shu, M. Zhang, B. Liu, Z. Wang, Enhancing desalination efficiency in PCM-FCDI systems through optimized interlayer channel design and flow dynamics, Desalination 600 (2025) 118478. https://doi.org/10.1016/j.desal.2024.118478.

[48] N.A. Thu Tran, N.M. Phuoc, H. Yoon, E. Jung, Y.-W. Lee, B.-G. Kang, H.S. Kang, C.-Y. Yoo, Y. Cho, Improved Desalination Performance of Flow- and Fixed-Capacitive Deionization using Redox-Active Quinone, ACS Sustain. Chem. Eng. 8 (2020) 16701–16710. https://doi.org/10.1021/acssuschemeng.0c06651.

[49] L. Bi, X. Sui, S. Tian, X. Liu, S. Ma, Z. Liu, Y. Liu, A. Ying, Semi-anchored percolation networks for low-threshold and selective desalination in FCDI, Desalination 616 (2025) 119417. https://doi.org/10.1016/j.desal.2025.119417.

[50] J. Ma, J. Ma, C. Zhang, J. Song, W. Dong, T.D. Waite, Flow-electrode capacitive deionization (FCDI) scale-up using a membrane stack configuration, Water Res. 168 (2020) 115186. https://doi.org/10.1016/j.watres.2019.115186.

[51] P. Liang, X. Sun, Y. Bian, H. Zhang, X. Yang, Y. Jiang, P. Liu, X. Huang, Optimized desalination performance of high voltage flow-electrode capacitive deionization by adding carbon black in flow-electrode, Desalination 420 (2017) 63–69. https://doi.org/10.1016/j.desal.2017.05.023.

[52] N. Minh Phuoc, N. Anh Thu Tran, T. Minh Khoi, H. Bin Jung, W. Ahn, E. Jung, C.-Y. Yoo, H.S. Kang, Y. Cho, ZIF-67 metal-organic frameworks and CNTs-derived nanoporous carbon structures as novel electrodes for flow-electrode capacitive deionization, Sep. Purif. Technol. 277 (2021) 119466. https://doi.org/10.1016/j.seppur.2021.119466.

[53] Y. Sun, Y. Cheng, F. Yu, J. Ma, Enhanced Salt Removal Performance Using Graphene-Modified Sodium Vanadium Fluorophosphate in Flow Electrode Capacitive Deionization, ACS Appl. Mater. Interfaces 13 (2021) 53850–53858. https://doi.org/10.1021/acsami.1c15205.

[54] B. Xie, Q. Liu, C. Hu, H. Li, G. Tan, D. Xiao, Enhanced desalination performance in flow electrode capacitive deionization with nitrogen doped porous carbon, New J. Chem. 47 (2023) 8625–8637. https://doi.org/10.1039/D3NJ00491K.

[55] X. Zhang, M. Pang, Y. Wei, F. Liu, H. Zhang, H. Zhou, Three-dimensional titanium mesh-based flow electrode capacitive deionization for salt separation and enrichment in high salinity water, Water Res. 251 (2024) 121147. https://doi.org/10.1016/j.watres.2024.121147.

[56] Z. Wang, X. Chen, Y. Zhang, J. Ma, Z. Lin, A. Abdelkader, M.-M. Titirici, L. Deng, Locally Enhanced Flow and Electric Fields Through a Tip Effect for Efficient Flow-Electrode Capacitive Deionization, Nano-Micro Lett. 17 (2025) 26. https://doi.org/10.1007/s40820-024-01531-0.

[57] K. Shen, Q. Wei, X. Wang, Q. Ru, X. Hou, G. Wang, K.S. Hui, J. Shen, K.N. Hui, F. Chen, Electrocatalytic desalination with CO_2_ reduction and O_2_ evolution, Nanoscale 13 (2021) 12157–12163. https://doi.org/10.1039/D1NR02578C.

[58] B. Kim, G. Piao, S. Kim, S.Y. Yang, Y. Park, D.S. Han, H.K. Shon, M.R. Hoffmann, H. Park, High-Efficiency Solar Desalination Accompanying Electrocatalytic Conversions of Desalted Chloride and Captured Carbon Dioxide, ACS Sustain. Chem. Eng. 7 (2019) 15320–15328. https://doi.org/10.1021/acssuschemeng.9b02640.

[59] H. Hu, M. Han, H. Lin, J. Dai, K. Shen, M. Li, X. Chen, A.Q. Zarifzoda, F. Liu, Y. Chen, F. Chen, Fast Seawater Desalination Integrated with Electrochemical CO_2_ Reduction, Angew. Chem. Int. Ed. 64 (2025) e202415806. https://doi.org/10.1002/anie.202415806.

[60] M. Li, W. Wang, M. Liang, Q. Yang, Y. Wang, L. Guo, Z. Yu, F. Chen, Y. Chen, Fast Seawater Desalination Driven by Efficient Nitrate Reduction via Bimetallic Iron/Zinc Polyphthalocyanine Frameworks, Angew. Chem. Int. Ed. (2025) e202506712. https://doi.org/10.1002/anie.202506712.

[61] M. Liang, W. Wang, M. Li, B. Luo, Q. Yang, L. Guo, K.N. Hui, G. Ying, F. Chen, Integrated electrochemical system using tin-phthalocyanine/graphene nanocomposite for synergistic formate production and rapid seawater desalination, J. Colloid Interface Sci. 703 (2026) 139178. https://doi.org/10.1016/j.jcis.2025.139178.

[62] A. Naderi Beni, S.M. Alnajdi, J. Garcia-Bravo, D.M. Warsinger, Semi-batch and batch low-salt-rejection reverse osmosis for brine concentration, Desalination 583 (2024) 117670. https://doi.org/10.1016/j.desal.2024.117670.

[63] S.K. Patel, P.M. Biesheuvel, M. Elimelech, Energy Consumption of Brackish Water Desalination: Identifying the Sweet Spots for Electrodialysis and Reverse Osmosis, ACS EST Eng. 1 (2021) 851–864. https://doi.org/10.1021/acsestengg.0c00192.

[64] C. Skuse, A. Gallego-Schmid, A. Azapagic, P. Gorgojo, Can emerging membrane-based desalination technologies replace reverse osmosis?, Desalination 500 (2021) 114844. https://doi.org/10.1016/j.desal.2020.114844.

[65] E. Bargiacchi, F. Orciuolo, L. Ferrari, U. Desideri, Use of Pressure-Retarded-Osmosis to reduce Reverse Osmosis energy consumption by exploiting hypersaline flows, Energy 211 (2020) 118969. https://doi.org/10.1016/j.energy.2020.118969.

[66] J. Kim, K. Park, D.R. Yang, S. Hong, A comprehensive review of energy consumption of seawater reverse osmosis desalination plants, Appl. Energy 254 (2019) 113652. https://doi.org/10.1016/j.apenergy.2019.113652.

[67] J.-K. Choi, A. Paudel, S. Sapkota, M. Alsehli, A. Alshamrani, J. Park, Y. Hong, X. Romeiko, Comparative assessment of conventional and emerging desalination technologies: a holistic review for sustainable water solutions, Desalination 614 (2025) 119140. https://doi.org/10.1016/j.desal.2025.119140.

[68] M. Hafiz, R. Alfahel, A. Altaee, A.H. Hawari, Techno-economic assessment of forward osmosis as a pretreatment process for mitigation of scaling in multistage flash seawater desalination process, Sep. Purif. Technol. 309 (2023) 123007. https://doi.org/10.1016/j.seppur.2022.123007.

[69] D. Dsilva Winfred Rufuss, V. Kapoor, S. Arulvel, P.A. Davies, Advances in forward osmosis (FO) technology for enhanced efficiency and output: A critical review, J. Clean. Prod. 356 (2022) 131769. https://doi.org/10.1016/j.jclepro.2022.131769.

[70] S. Liyanaarachchi, V. Jegatheesan, L. Shu, H.K. Shon, S. Muthukumaran, C.Q. Li, Evaluating the Feasibility of Forward Osmosis in Diluting RO Concentrate Using Pretreatment Backwash Water, Membranes 10 (2020) 35. https://doi.org/10.3390/membranes10030035.

[71] A. Al-Karaghouli, L.L. Kazmerski, Energy consumption and water production cost of conventional and renewable-energy-powered desalination processes, Renew. Sustain. Energy Rev. 24 (2013) 343–356. https://doi.org/10.1016/j.rser.2012.12.064.

[72] Q. Chen, M. Burhan, K.J. M., Y. Li, K.C. Ng, An ocean thermocline desalination system using the direct spray method, Desalination 520 (2021) 115373. https://doi.org/10.1016/j.desal.2021.115373.

[73] K. Al Bkoor Alrawashdeh, K.K. Al-Zboon, L.A. Al-samrraie, R. Momani, T. Momani, E. Gul, P. Bartocci, F. Fantozzi, Performance of dual multistage flashing - recycled brine and solar power plant, in the framework of the water-energy nexus, Energy Nexus 5 (2022) 100046. https://doi.org/10.1016/j.nexus.2022.100046.

[74] A.M.K. El-Ghonemy, Performance test of a sea water multistage flash distillation plant: Case study, Alex. Eng. J. 57 (2018) 2401–2413. https://doi.org/10.1016/j.aej.2017.08.019.

[75] Z. Hu, Y. Chen, Advancements in sustainable desalination with ocean thermal energy: A review, Desalination 586 (2024) 117770. https://doi.org/10.1016/j.desal.2024.117770.

[76] P. Ellersdorfer, A. Omar, R.A. Taylor, R. Daiyan, G. Leslie, Multi-effect distillation: a sustainable option to large-scale green hydrogen production using solar energy, Int. J. Hydrog. Energy 48 (2023) 31491–31505. https://doi.org/10.1016/j.ijhydene.2023.04.261.

[77] M. Tayefeh, An innovative rearrangement and comprehensive comparison of the combination of compressed air energy storage (CAES) with multi stage flash (MSF) desalination and multi effect distillation (MED) systems, J. Energy Storage 52 (2022) 105025. https://doi.org/10.1016/j.est.2022.105025.

[78] M. Prajapati, M. Shah, B. Soni, A comprehensive review of the geothermal integrated multi-effect distillation (MED) desalination and its advancements, Groundw. Sustain. Dev. 19 (2022) 100808. https://doi.org/10.1016/j.gsd.2022.100808.

[79] A. Chorak, P. Palenzuela, D.-C. Alarcón-Padilla, A. Ben Abdellah, Experimental characterization of a multi-effect distillation system coupled to a flat plate solar collector field: Empirical correlations, Appl. Therm. Eng. 120 (2017) 298–313. https://doi.org/10.1016/j.applthermaleng.2017.03.115.

[80] S. Noamani, S. Niroomand, M. Rastgar, M. Azhdarzadeh, M. Sadrzadeh, Modeling of Air-Gap Membrane Distillation and Comparative Study with Direct Contact Membrane Distillation, Ind. Eng. Chem. Res. 59 (2020) 21930–21947. https://doi.org/10.1021/acs.iecr.0c04464.

[81] A.A. Khan, L.N. Nthunya, S. Farooq, I.A. Khan, M. Zargar, B.B. Mamba, W. Suwaileh, Review of fluoropolymer-based membranes for membrane distillation applications, Chem. Eng. Res. Des. 223 (2025) 717–737. https://doi.org/10.1016/j.cherd.2025.10.039.

[82] Y. Soumbati, I. Bouatou, A. Abushaban, Y. Belmabkhout, M.C. Necibi, Review of membrane distillation for desalination applications: Advanced modeling, specific energy consumption, and water production cost, J. Water Process Eng. 71 (2025) 107296. https://doi.org/10.1016/j.jwpe.2025.107296.

[83] R. Schwantes, A. Cipollina, F. Gross, J. Koschikowski, D. Pfeifle, M. Rolletschek, V. Subiela, Membrane distillation: Solar and waste heat driven demonstration plants for desalination, Desalination 323 (2013) 93–106. https://doi.org/10.1016/j.desal.2013.04.011.

[84] A. Najim, A review of advances in freeze desalination and future prospects, Npj Clean Water 5 (2022) 15. https://doi.org/10.1038/s41545-022-00158-1.

[85] S. Yi, Q. Gao, J. Song, H. Wang, H. Yuan, Experimental study on seawater freeze desalination based on ultrasonic vibration, Desalination 596 (2025) 118336. https://doi.org/10.1016/j.desal.2024.118336.

[86] K.J. Lu, Z.L. Cheng, J. Chang, L. Luo, T.-S. Chung, Design of zero liquid discharge desalination (ZLDD) systems consisting of freeze desalination, membrane distillation, and crystallization powered by green energies, Desalination 458 (2019) 66–75. https://doi.org/10.1016/j.desal.2019.02.001.

[87] P. Wu, X. Wu, Y. Wang, H. Xu, G. Owens, Towards sustainable saline agriculture: Interfacial solar evaporation for simultaneous seawater desalination and saline soil remediation, Water Res. 212 (2022) 118099. https://doi.org/10.1016/j.watres.2022.118099.

[88] Y. Ko, S. Lee, J. Jang, G. Kwon, K. Lee, Y. Jeon, A. Lee, T. Park, J. Kim, J. You, Nanocellulose‐Based Interfacial Solar Evaporator: Integrating Sustainable Materials and Micro‐/Nano‐Architectures for Solar Desalination, Adv. Funct. Mater. 35 (2025) 2414576. https://doi.org/10.1002/adfm.202414576.

[89] N.T. Cuong, N. Van Canh, N.H. Hoa, T.H. Pham, H.T. Pham, L.T.H. Phong, C.T.A. Tuyet, N.T.N. Hang, V.-D. Dao, V. Nguyen, Tilted 3D evaporator with high-performance salt rejection for seawater desalination, Desalination 574 (2024) 117303. https://doi.org/10.1016/j.desal.2024.117303.

[90] Y. Li, J. Styczynski, Y. Huang, Z. Xu, J. McCutcheon, B. Li, Energy-positive wastewater treatment and desalination in an integrated microbial desalination cell (MDC)-microbial electrolysis cell (MEC), J. Power Sources 356 (2017) 529–538. https://doi.org/10.1016/j.jpowsour.2017.01.069.

[91] X. Cao, X. Huang, P. Liang, K. Xiao, Y. Zhou, X. Zhang, B.E. Logan, A New Method for Water Desalination Using Microbial Desalination Cells, Environ. Sci. Technol. 43 (2009) 7148–7152. https://doi.org/10.1021/es901950j.

[92] M. Khazraee Zamanpour, H.-R. Kariminia, M. Vosoughi, Electricity generation, desalination and microalgae cultivation in a biocathode-microbial desalination cell, J. Environ. Chem. Eng. 5 (2017) 843–848. https://doi.org/10.1016/j.jece.2016.12.045.

[93] S. Rahman, Tahereh Jafary, A. Al-Mamun, M.S. Baawain, M.R. Choudhury, H. Alhaimali, S.A. Siddiqi, B.R. Dhar, A. Sana, S.S. Lam, M. Aghbashlo, M. Tabatabaei, Towards upscaling microbial desalination cell technology: A comprehensive review on current challenges and future prospects, J. Clean. Prod. 288 (2021) 125597. https://doi.org/10.1016/j.jclepro.2020.125597.

[94] C. Du, J.R. Du, X. Zhao, F. Cheng, M.E.A. Ali, X. Feng, Treatment of Brackish Water RO Brine via Bipolar Membrane Electrodialysis, Ind. Eng. Chem. Res. 60 (2021) 3115–3129. https://doi.org/10.1021/acs.iecr.1c00370.

[95] C.G. Patel, J. Swaminathan, Flowpath modification to reduce electrodialysis module size and energy consumption, Sep. Purif. Technol. 362 (2025) 131555. https://doi.org/10.1016/j.seppur.2025.131555.

[96] S.K. Patel, P.M. Biesheuvel, M. Elimelech, Energy Consumption of Brackish Water Desalination: Identifying the Sweet Spots for Electrodialysis and Reverse Osmosis, ACS EST Eng. 1 (2021) 851–864. https://doi.org/10.1021/acsestengg.0c00192.

[97] M. Turek, Cost effective electrodialytic seawater desalination, Desalination 153 (2003) 371–376. https://doi.org/10.1016/S0011-9164(02)01130-X.

[98] C.C.N. Kunrath, D.C. Patrocínio, M.A. Siqueira Rodrigues, T. Benvenuti, F.D.R. Amado, Electrodialysis reversal as an alternative treatment for producing drinking water from brackish river water: A case study in the dry season, northeastern Brazil, J. Environ. Chem. Eng. 8 (2020) 103719. https://doi.org/10.1016/j.jece.2020.103719.

[99] T. Kim, C.A. Gorski, B.E. Logan, Low Energy Desalination Using Battery Electrode Deionization, Environ. Sci. Technol. Lett. 4 (2017) 444–449. https://doi.org/10.1021/acs.estlett.7b00392.

[100] H. Wang, X. Xu, X. Gao, Y. Li, T. Lu, L. Pan, Design of three-dimensional faradic electrode materials for high-performance capacitive deionization, Coord. Chem. Rev. 510 (2024) 215835. https://doi.org/10.1016/j.ccr.2024.215835.

[101] A. Fombona-Pascual, N. Patil, E. García-Quismondo, N. Goujon, D. Mecerreyes, R. Marcilla, J. Palma, J.J. Lado, A high performance all-polymer symmetric faradaic deionization cell, Chem. Eng. J. 461 (2023) 142001. https://doi.org/10.1016/j.cej.2023.142001.

[102] Z. Hao, X. Sun, J. Chen, X. Zhou, Y. Zhang, Recent Progress and Challenges in Faradic Capacitive Desalination: From Mechanism to Performance, Small 19 (2023) 2300253. https://doi.org/10.1002/smll.202300253.

[103] Z.G. Neale, R.H. DeBlock, M.E. Tighe, D.R. Rolison, J.W. Long, Architected Silver Sponges for Faradaic Deionization of Salt Water, (2024). https://doi.org/10.2139/ssrn.4938315.

[104] P. Lin, R. Yu, Y. Wang, T. Yang, Z. Li, J. Zhang, X. Yi, Z. Liu, X. Xu, Mechanism insight into improved desalination performance and energy efficiency in redox flow deionization with ion exchange resins, Chem. Eng. J. 475 (2023) 145940. https://doi.org/10.1016/j.cej.2023.145940.

[105] P. Lin, T. Yang, Z. Li, W. Xia, X. Xuan, X. Sun, S.M. Alshehri, T. Ahamad, Y. Yamauchi, X. Xu, Y. Bando, Ion transport channels in redox flow deionization enable ultra-high desalination performance, Nano Energy 102 (2022) 107652. https://doi.org/10.1016/j.nanoen.2022.107652.

[106] B. Kim, J.Y. Seo, C.-H. Chung, A hybrid system of capacitive deionization and redox flow battery for continuous desalination and energy storage, J. Power Sources 448 (2020) 227384. https://doi.org/10.1016/j.jpowsour.2019.227384.

[107] D. Ahn, D. Kim, J.H. Park, N. Kim, E. Lim, C. Kim, Enhanced desalination performance of nitrogen-doped porous carbon electrode in redox-mediated deionization, Desalination 520 (2021) 115333. https://doi.org/10.1016/j.desal.2021.115333.

[108] H. Kim, S. Kim, N. Kim, X. Su, C. Kim, Multi-electrode scale-up strategy and parametric investigation of redox-flow desalination systems, Desalination 549 (2023) 116350. https://doi.org/10.1016/j.desal.2022.116350.

[109] K. Tang, Y. Kim, J. Chang, R.T. Mayes, J. Gabitto, S. Yiacoumi, C. Tsouris, Seawater desalination by over-potential membrane capacitive deionization: Opportunities and hurdles, Chem. Eng. J. 357 (2019) 103–111. https://doi.org/10.1016/j.cej.2018.09.121.

[110] S. Kumar, N.M. Aldaqqa, E. Alhseinat, D. Shetty, Electrode Materials for Desalination of Water via Capacitive Deionization, Angew. Chem. 135 (2023) e202302180. https://doi.org/10.1002/ange.202302180.

[111] J. Ma, C. Zhai, F. Yu, Review of flow electrode capacitive deionization technology: Research progress and future challenges, Desalination 564 (2023) 116701. https://doi.org/10.1016/j.desal.2023.116701.

[112] C. Santos, I.V. Rodríguez, J.J. Lado, M. Vila, E. García-Quismondo, M.A. Anderson, J. Palma, J.J. Vilatela, Low-energy consumption, free-form capacitive deionization through nanostructured networks, Carbon 176 (2021) 390–399. https://doi.org/10.1016/j.carbon.2021.01.148.

[113] S. Yang, H. Kim, S. Jeon, J. Choi, J. Yeo, H. Park, J. Jin, D.K. Kim, Analysis of the desalting performance of flow-electrode capacitive deionization under short-circuited closed cycle operation, Desalination 424 (2017) 110–121. https://doi.org/10.1016/j.desal.2017.09.032.

[114] C. Kim, P. Srimuk, J. Lee, M. Aslan, V. Presser, Semi-continuous capacitive deionization using multi-channel flow stream and ion exchange membranes, Desalination 425 (2018) 104–110. https://doi.org/10.1016/j.desal.2017.10.012.

[115] B. Halder, Md.M. Hossen, Md.S. Hossain, M. Mourshed, Toward scalable and energy efficient desalination: Synergistic advances in FCDI design, Energy Rep. 14 (2025) 4750–4765. https://doi.org/10.1016/j.egyr.2025.11.084.

[116] C. Zhang, J. Ma, L. Wu, J. Sun, L. Wang, T. Li, T.D. Waite, Flow Electrode Capacitive Deionization (FCDI): Recent Developments, Environmental Applications, and Future Perspectives, Environ. Sci. Technol. 55 (2021) 4243–4267. https://doi.org/10.1021/acs.est.0c06552.

[117] J. Lim, S. Lee, H. Lee, S. Hong, Energetic Comparison of Flow-Electrode Capacitive Deionization and Membrane Technology: Assessment on Applicability in Desalination Fields, Environ. Sci. Technol. 58 (2024) 6181–6191. https://doi.org/10.1021/acs.est.4c00672.

[118] R. McNair, G. Szekely, R.A.W. Dryfe, Ion-Exchange Materials for Membrane Capacitive Deionization, ACS EST Water 1 (2021) 217–239. https://doi.org/10.1021/acsestwater.0c00123.

[119] J.-H. Choi, D.-J. Yoon, A stable operation method for membrane capacitive deionization systems without electrode reactions at high cell potentials, Water Res. 157 (2019) 167–174. https://doi.org/10.1016/j.watres.2019.03.083.

[120] S. Porada, L. Zhang, J.E. Dykstra, Energy consumption in membrane capacitive deionization and comparison with reverse osmosis, Desalination 488 (2020) 114383. https://doi.org/10.1016/j.desal.2020.114383.

[121] T.-H. Chen, Y.-H. Kao, Y.-Y. Shen, C.-S. Fan, C.-H. Hou, Pilot-scale membrane capacitive deionization for water reclamation: Commissioning, performance benchmarking, and long-term assessment, Desalination 599 (2025) 118428. https://doi.org/10.1016/j.desal.2024.118428.

[122] C. Tan, C. He, J. Fletcher, T.D. Waite, Energy recovery in pilot scale membrane CDI treatment of brackish waters, Water Res. 168 (2020) 115146. https://doi.org/10.1016/j.watres.2019.115146.

[123] M. Elimelech, W.A. Phillip, The Future of Seawater Desalination: Energy, Technology, and the Environment, Science 333 (2011) 712–717. https://doi.org/10.1126/science.1200488.

[124] Z. Jiang, S. Ren, X. Cao, Q. Fan, R. Yu, J. Yang, J. Mao, pH‐Universal Electrocatalytic CO_2_ Reduction with Ampere‐level Current Density on Doping‐engineered Bismuth Sulfide, Angew. Chem. Int. Ed. 63 (2024) e202408412. https://doi.org/10.1002/anie.202408412.

[125] C.P. O’Brien, R.K. Miao, A. Shayesteh Zeraati, G. Lee, E.H. Sargent, D. Sinton, CO_2_ Electrolyzers, Chem. Rev. 124 (2024) 3648–3693. https://doi.org/10.1021/acs.chemrev.3c00206.

[126] S. Jin, Z. Hao, K. Zhang, Z. Yan, J. Chen, Advances and Challenges for the Electrochemical Reduction of CO_2_ to CO: From Fundamentals to Industrialization, Angew. Chem. Int. Ed. 60 (2021) 20627–20648. https://doi.org/10.1002/anie.202101818.

[127] W. Deng, A. Lee, W. Dai, L. Cherniack, B.S. Crandall, H. Li, F. Jiao, Techno-economics of polymer-membrane-based CO_2_ electrolysers, Nat. Rev. Clean Technol. 1 (2025) 255–268. https://doi.org/10.1038/s44359-025-00045-1.

[128] D. Choi, J. Kim, S. Jaffer, B. Chen, K. Xie, E.H. Sargent, Translating insights from progress in photovoltaics to accelerate industrial-scale CO_2_ electroreduction, Nat. Energy 11 (2026) 185–193. https://doi.org/10.1038/s41560-025-01953-z.

[129] R. Schwantes, Y. Morales, E. Pomp, J. Singer, K. Chavan, F. Saravia, Thermally driven ultrapure water production for water electrolysis – A techno-economic analysis of membrane distillation, Desalination 608 (2025) 118848. https://doi.org/10.1016/j.desal.2025.118848.
